# Long-distance communication can enable collective migration in a dynamic seascape

**DOI:** 10.1038/s41598-024-65827-2

**Published:** 2024-06-27

**Authors:** Stephanie Dodson, William K. Oestreich, Matthew S. Savoca, Elliott L. Hazen, Steven J. Bograd, John P. Ryan, Jerome Fiechter, Briana Abrahms

**Affiliations:** 1 Department of Mathematics, Colby College, Waterville, ME 04901 USA; 2 Monterey Bay Aquarium Research Institute, Moss Landing, CA 95039 USA; 3 Hopkins Marine Station, Department of Biology, Stanford University, Pacific Grove, CA 93950 USA; 4 Environmental Research Division, Southwest Fisheries Science Center, National Oceanic and Atmospheric Administration, Monterey, CA 93940 USA; 5 Institute of Marine Sciences, University of California, Santa Cruz, Santa Cruz, CA 95064 USA; 6 Department of Ocean Sciences, University of California Santa Cruz, Santa Cruz, CA 95064 USA; 7 Department of Biology, Center for Ecosystem Sentinels, University of Washington, Seattle, WA 98195 USA

**Keywords:** Migration, Collective behavior, Social information, Individual-based model, Resource tracking, Pelagic, Blue whale (*Balaenoptera musculus*), Krill, Behavioural ecology, Biooceanography

## Abstract

Social information is predicted to enhance the quality of animals’ migratory decisions in dynamic ecosystems, but the relative benefits of social information in the long-range movements of marine megafauna are unknown. In particular, whether and how migrants use nonlocal information gained through social communication at the large spatial scale of oceanic ecosystems remains unclear. Here we test hypotheses about the cues underlying timing of blue whales’ breeding migration in the Northeast Pacific via individual-based models parameterized by empirical behavioral data. Comparing emergent patterns from individual-based models to individual and population-level empirical metrics of migration timing, we find that individual whales likely rely on both personal and social sources of information about forage availability in deciding when to depart from their vast and dynamic foraging habitat and initiate breeding migration. Empirical patterns of migratory phenology can only be reproduced by models in which individuals use long-distance social information about conspecifics’ behavioral state, which is known to be encoded in the patterning of their widely propagating songs. Further, social communication improves pre-migration seasonal foraging performance by over 60% relative to asocial movement mechanisms. Our results suggest that long-range communication enhances the perceptual ranges of migrating whales beyond that of any individual, resulting in increased foraging performance and more collective migration timing. These findings indicate the value of nonlocal social information in an oceanic migrant and suggest the importance of long-distance acoustic communication in the collective migration of wide-ranging marine megafauna.

## Introduction

Across the animal kingdom, diverse taxa undertake long-distance movements to track the availability of resources in space and time^1^. The ability of migrating animals to match their timing and locations with optimal resource availability has critical consequences for individual energy gain, fitness, and population persistence^2–4^. The sources of information that animals rely on to make these movement decisions can determine how migratory populations respond to environmental variability^5^, how they find and exploit prey resources^6^, and how they fare under rapid environmental change^7,8^. As a result, considerable research has sought to understand the cues that animals use to decide when and where to move and migrate^5,9–11^. Nevertheless, which mechanisms guide long-distance migratory behavior remains a key question in ecology and this understanding is critical for anticipating the impacts of rapid environmental change on threatened migratory populations.

A range of cues, both internal and external and spanning fixed to dynamic, influence migratory decisions^5,12^. Many migratory animals rely on a range of information sources acquired via personal experience^13–15^ and/or social cues^9,11,16,17^. The relative utility of these information sources varies with life history traits, habitat complexity and predictability, and social context. For example, long-lived species may rely heavily on memory and learning gained through individual experience^13,18,19^. Moreover, the relative utility of personal information depends on perceptual range, as nonlocal information allows the assessment of environmental variation over broader spatial scales in heterogeneous environments^15^.

Beyond individual sensing and learning, social information can play an important role in migratory decisions^9,11,16^. This social information—including both intentional signals and inadvertent cues among individuals^14,20^—can greatly enhance access to nonlocal information by expanding perceptual ranges well beyond that of any individual alone^21^. Such information transfer can enable individuals to detect noisy environmental gradients and make better-informed migratory decisions, improving the ability to collectively track favorable resources or avoid predation risk when migrating^10,11,22^. While such collective migrations are increasingly recognized throughout the animal kingdom, theory on collective sensing and migration has primarily been developed and tested via study of social species which migrate in groups (e.g., salmon^23^ and storks^24^). Such theory has highlighted the importance of group size^25,26^ and density^23,27^ in allowing for emergent sensing and collective behaviors including migration. Yet many species can communicate over substantial distance, indicating that collective migrations might emerge even in low-density populations^22^ and enable collective sensing of nonlocal information over even broader spatial scales than possible by aggregated groups. Theoretical simulations suggest that access to such nonlocal information should provide particular benefit in systems characterized by steep resource gradients and ephemeral resource patches^15^, yet tests of this hypothesis in the context of migration remain sparse.

The pelagic ocean represents the vast majority of Earth’s habitable space and is characterized by dynamism and patchiness across spatial and temporal scales^28,29^. In dynamic oceanic ecosystems, resources are extremely non-uniformly distributed, aggregating in ephemeral hotspots^30–32^. These attributes suggest that nonlocal information should provide particularly valuable information for pelagic animals’ long-distance foraging and migratory movements, yet whether and how migrants use nonlocal social information at the extreme spatial scale of oceanic ecosystems is unclear. Making detailed and persistent behavioral observations across ecological scales in vast and largely opaque oceanic ecosystems has been challenging historically, and has limited the ability to test whether nonlocal social information gives rise to collective migrations in the pelagic. We address this question using multi-year, continuous empirical observations of population-level foraging and migration timing in blue whales (*Balaenoptera musculus*) in comparison to empirically parameterized models of prey distribution, oceanographic conditions, and individual whale movement and communication.

Blue whales are obligatory krill predators and require significant prey resources to support their enormous body size^33^. In the Northeast Pacific Ocean, blue whales migrate between seasonally productive foraging grounds off the west coast of North America in summer and fall, and breeding grounds in the Gulf of California and west of Central America during winter and spring^34^. The summer-fall foraging grounds represent the primary source of annual energy acquisition for individuals in this population^35^. These foraging grounds in the highly productive California Current system display considerable intra- and inter-annual variation. Within the foraging season, blue whales track their obligate krill prey that aggregate in ephemeral oceanographic habitats such as regions of enhanced upwelling or surface convergence^36–39^. These fine-scale resource tracking strategies allow an adult blue whale to consume ~ 1500 tonnes of krill during the foraging season in the California Current^40^, which fuels their long-distance migration to and from wintering grounds and rearing of young^35^.

It is unknown how blue whales decide when to depart from these foraging grounds and initiate their southward breeding migration. Previous research has documented that blue whales track the long-term average ecosystem phenology as they migrate northward towards the feeding grounds^13^. This strategy of reliance on memory over contemporaneous environmental cues maximizes their likelihood of energy acquisition in the long term (between years and over decades). In contrast, blue whales track contemporaneous ecosystem conditions when departing the foraging habitat, shifting the population-level timing of migration by as much as four months to match year-to-year variation in the phenology of upwelling that drives krill prey abundance^41^. But what signals or cues enable this plasticity in foraging and migration timing in such a vast and dynamic habitat?

Detecting the change in behavioral state of this blue whale population, from foraging to migration, is possible by evaluating the diel patterns of song production at ecosystem scale^42^. Song production in blue whales has often been associated with reproduction as it is believed to be a male-specific behavior^43^. Yet seasonal and diel patterns of song production indicate that information about behavioral state is also encoded in blue whales’ songs. During the foraging months, blue whales in this population sing primarily at night due to intensive daytime foraging effort, but transition to a more even diel song distribution following the cessation of foraging and onset of breeding migration^42^. These diel patterns enable researchers to track population-wide behavioral transitions throughout the Northeast Pacific^41,44^, prompting the question of whether blue whales themselves use long-distance information about conspecifics’ behavioral state to inform their migratory decisions.

**Figure 1:**
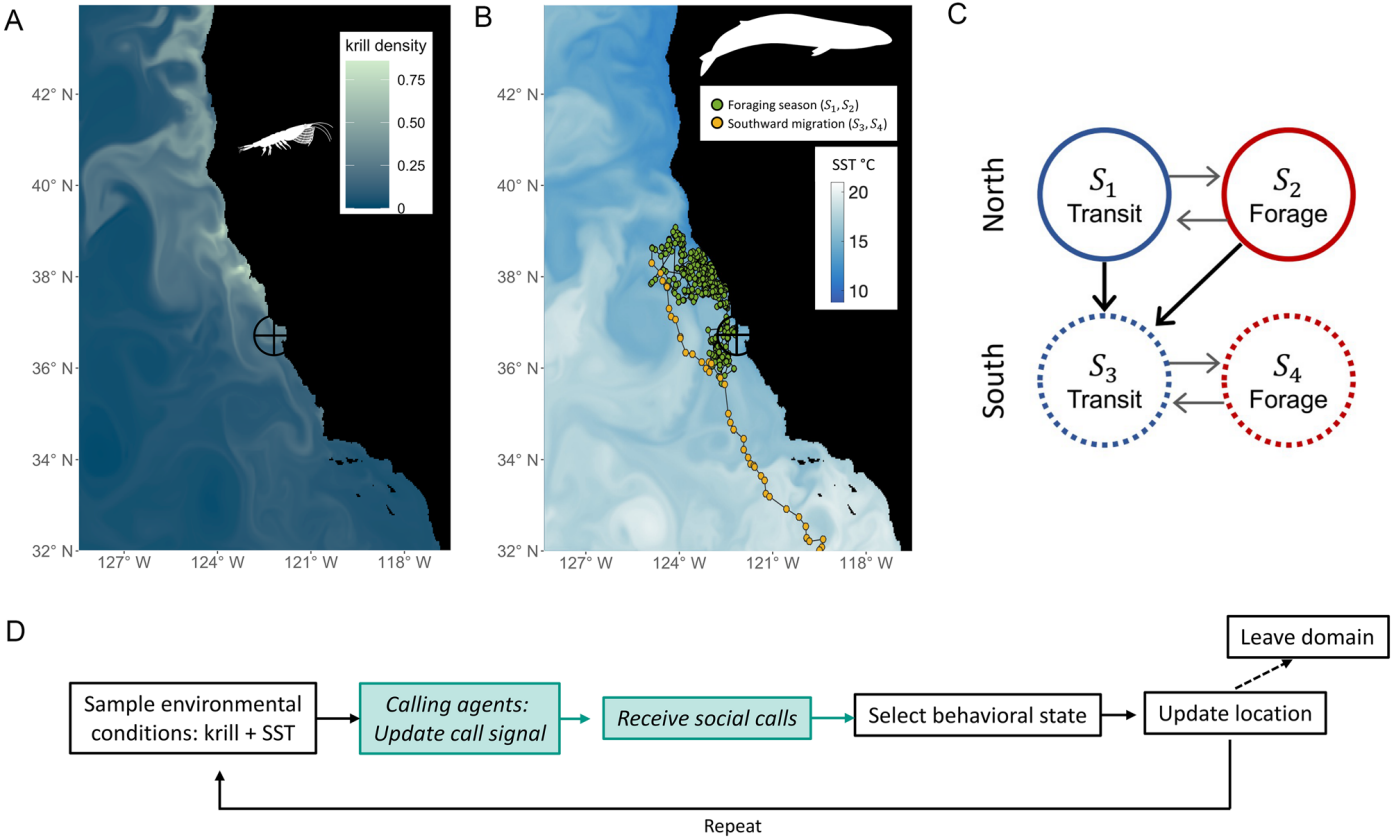
Model schematic. (**A**) Example of simulated near-surface krill density over the simulated domain on yearday 250 (2004). (**B**) Example of a simulated blue whale track, with each colored circle indicating this individual’s position and behavioral state at each 6-h time interval. This individual exited the simulation domain on yearday 310 (2004), the day for which simulated sea surface temperature (SST) is shown. In both (**A,B**), the larger black circle indicates the approximate blue whale song detection range of the Monterey Bay hydrophone^42^, which is located at the cross intersection within the circle. (**C**) Behavioral states in the IBM, with arrows indicate possible behavioral state transitions. “North” states (*S*_1_ and* S*_2_) refer to transiting and foraging states on higher-latitude foraging grounds before the initiation of the southward breeding migration. “South” states (*S*_3_ and *S*_4_) refer to these states after the initiation of the southward breeding migration. (**D**) Flow chart describing the model process for each simulated agent at each 6-h time step of the simulation. Blue boxes in this flow chart are only included in the version of the simulation which includes use of social information (*m*_*per & soc*_; see Table 1 for details). For a complete description of the model formulation and sensitivity analysis, see Supplementary Figs. 1–4. Maps in (**A,B**) generated using Matlab R2022b (https://www.mathworks.com/) and R version 4.2.0 (https://www.r-project.org/).

Individual-based models (IBMs) are a modeling framework that can be used to examine how individual-based movement mechanisms generate population-level patterns. In this study, we encode a series of IBMs to investigate how population-level breeding migration behaviors emerge from hypothesized short-term foraging and migration decisions based on recent personal foraging experiences and/or social information gained from long-distance communication (Fig. 1, Table 1). We compare empirically observed patterns of population-level migratory behavior to those emerging from the IBMs to deduce which of these decision mechanisms best replicates empirical observations. Using this integrative approach, we assess two related hypotheses (Table 2) concerning (i) the information sources on which whales rely in deciding when to depart from foraging habitat and begin their equatorward migration; and (ii) the consequences of reliance on different cue types for seasonal foraging performance. The results of this study yield insight about the cues underlying blue whales’ migratory decisions. More broadly, this approach elucidates the dynamics of collective migration in this widely distributed, low-density population, a space-use pattern found in populations across the animal kingdom.

**Table 1:** Summary of tested model behavioral states and factors included in southward migration state transition function.

*m*_0_*:* non-migratory (null)	*m*_*per*_*:* personal information	*m*_*per* + *soc*_*:* personal + social information
2 states: *S*_1,2_	4 states: *S*_1,2,3,4_	4 states: *S*_1,2,3,4_
No transition to southward migration	Transition to southward migration dependent on individual foraging efficiency	Transition to southward migration dependent on both individual foraging efficiency and the behavioral state of calling conspecifics within acoustic detection range

**Table 2:** Summary of metrics used to assess questions and hypotheses concerning the influence of personal and social cues on population-level migration timing and seasonal foraging performance.

Timing of migration	Seasonal foraging performance
Q_1_: What behavioral cues lead to realistic southward migration timing?	Q_2_: What behavioral cues maximize population-level krill intake?
H_1_: Personal information alone (*m*_*per*_) is insufficient to enable realistically timed and interannually flexible southward migrations. Realistic migration timing and flexibility arises only when social foraging information is considered (*m*_per & soc_)	H_2_: Krill intake is highest when both social foraging information is considered (*m*_*per & soc*_)
Assessment metric: Population-level migration timing from each model compared to empirical measurements^41,50^	Assessment metric: Total annual krill intake relative to *m*_0_

## Methods

### Environmental and prey data

Environmental conditions, specifically sea surface temperature (SST) and near-surface (0–200 m) krill abundance, are provided from a Regional Ocean Modeling System (ROMS) implementation that has been coupled with a biogeochemical model, NEMUCSC, adapted from the North Pacific Ecosystem Model for Understanding Regional Oceanography of Kishi et al.^45^ and specifically parameterized for the California Current region^46,47^. The model provides 3 km spatial and daily temporal resolution of SST and krill concentrations over a study domain of 116–128° W and 32–44° N for the years 1990–2010^46^; simulated krill concentrations have been evaluated against existing in situ data for May–June and are known to accurately represent spatial (mesoscale) and temporal (intraseasonal and interannual) variability in observed krill aggregation regions during the upwelling season^46^. The ROMS-NEMUCSC output is pre-computed and supplied as input to the IBMs.

### Hydrophone data and empirical social communications

Empirical studies^41,42,44^ found that blue whales’ population-level song call production increases throughout the foraging season in the Northeast Pacific, typically peaking from October through November. Only male blue whales have been recorded producing song calls in the Northeast Pacific^43,48^. Furthermore, the proportion of song produced during the night versus the day generally increases through at least September, then shows a drop toward a more even diel distribution of song. The timing of this drop in the proportion of song produced at night varies﻿ interannually (August–January^41^) and has been attributed to the population-level transition from foraging to southward migration^42^. Because Northeast Pacific blue whales forage primarily during the day and foraging and singing behavior are temporally separate at sub-daily scale, predominantly nighttime singing behavior is strongly correlated with foraging behavior. As a result, the 24-h patterning of blue whale song effectively provides long-distance information on the behavioral state of conspecifics in a vast and dynamic foraging arena^42^. While the precise range over which blue whales can detect conspecifics’ songs remains uncertain, hydrophones can detect these songs at high signal-to-noise ratio over ranges exceeding 100 km^42^. CT scanning and modeling-based efforts quantifying closely related fin whale (*B. physalus*) hearing sensitivity^49^ indicate auditory sensitivity of ~ 100 dB re 1 μPa in the frequency range of blue whale songs, which would allow for blue whale song detection over at least tens of km in Monterey Bay, CA, USA^42^.

Data from the Monterey Accelerated Research System hydrophone (hereafter “Monterey Bay hydrophone”^41,42^) and tag analysis^50^ reveal that median blue whale southward breeding migration dates most often fall in October through early November. High year-to-year variation is present with median departure dates ranging from mid-September to mid-December. Migrations for individual blue whales are recorded from as early as August to as late as January^41,50^.

### Individual-based model framework

Individual-based models (IBMs) are a flexible model type that treat individuals as autonomous agents who follow a set of individual-level decisions and updates. Thus, IBMs are well suited to test how various strategies at the individual-level develop into population-level emergent behaviors, such as migrations. Comparing population-level behaviors of IBMs with empirical data provides insight into which short-term decisions contribute to observed broad-scale behaviors. IBMs have previously been used to understand the spatiotemporal movement of populations, including migrations of pelagic fish^51^, resource-driven formation of locust bands^52^, and group formation of zebrafish during movement^53^.

We provide an overview of the model here; full model details are available in the Supplement, including a description of the overview, design concepts, and details (ODD) framework, a protocol for standardizing published descriptions of IBMs^54,55^. The flow chart in Fig. 1D provides a summary of the core model processes and sequences. Our IBM is formatted as a probabilistic state-switching model that treats individual whales as autonomous agents that move around the two-dimensional domain and are faced with decisions including foraging, transiting, or migrating south at each discrete time step. Dodson et al.^56^ presented a series of IBMs with probabilistic state transitions based only on sea-surface temperature (SST) and krill densities. These models accurately captured early-season foraging patterns of Eastern North Pacific blue whales but lacked inter-agent communication and exhibited unrealistically early breeding migration timings. The IBM presented here is an adaptation and extension of those parameterized in Ref.^56^ with the goal of focusing on southward breeding migration strategies.

Four behavioral states are defined and implemented in the IBM (Fig. 1C) that characterize observed behaviors of Eastern North Pacific blue whales. Previous analysis of tag data revealed and parameterized distinct short-term movement distributions in blue whales associated with transit and forage behaviors^34^ throughout the foraging season. Furthermore, blue whales have been observed to definitively switch into southward migration behaviors, marked by an end in foraging and onset of intentional southward movement, sometimes with occasional pauses to forage^42^. Therefore, modeled behavioral states *S*_1,2_ represent transit and foraging behaviors, respectively, during the summer to fall high-latitude foraging season and *S*_3,4_ represent transit and forage states for the southward breeding migration. Each state *S*_*k*_ is associated with distinct movement updates that capture the known behavioral patterns. We utilize the previously defined movement distributions^34,56^ and transit-forage decisions (based on SST and krill)^56^ that allow within-season transitions (transitions from *S*_1_ to *S*_2_ and *S*_3_ to *S*_4_) and we test several migration transition strategies that initiate the southward breeding migration (transitions from *S*_1,2_ to *S*_3_).

In summary, the model proceeds as follows (see schematic in Fig. 1D). At every time-step (6-h intervals), each agent uses a combination of local forage conditions (SST and krill concentrations from ROMS-NEMUCSC), personal foraging experiences, and (in model runs including use of social information) received social information to select a behavioral state with probabilities defined by a state transition matrix. A movement update (turning angle and step length) is selected from the designated behavioral state movement distribution, and the agent’s position is updated. To focus on factors driving the breeding migration, yearly models are initiated on July 1st with agents’ initial locations selected uniformly at random from areas of climatologically high krill densities from the ROMS-NEMUCSC solution^13^. Throughout the model description, a subscript $n$ indicates variables and quantities specific for agent *n*. Time dependence is indicated with *t*, corresponding to the time step.

### Model implementation of social communication

Inter-agent communication is implemented by dividing communication into the subprocesses of call production and reception. Half of the agents are randomly initialized as male song producers, and a proportion of male agents are randomly selected to call at each time-step. The proportion of calling male agents increases linearly throughout the season from 5 to 30%, following the empirically observed trend of increasing received call intensities detected by the Monterey Bay hydrophone over summer and fall^42^. The IBM uses daily mean simulated prey and SST data and does not distinguish night versus day. Instead, we define ${\overline{x} }_{n}(t)$ to be the average proportion of time spent foraging of agent *n* during the past 24 h and define the call signal by ${\delta }_{n}(t)=\text{tanh}[{b}_{1} ({\overline{x} }_{n}(t)-{b}_{2})].$ The call signal ${\delta }_{n}(t)$ transforms ${\overline{x} }_{n}(t)$ into a value between − 1 and 1, with positive (negative) values indicating individual *n* spent more time foraging (transiting) during the time-period. The sign and value of ${\delta }_{n}(t)$ is intended to mimic the diel patterns in hydrophone recordings of blue whale songs.

All agents act as call receivers. Individual agents receive and average call signals from nearby individuals; call signals ${\delta }_{n}(t)$ are weighted with a distance-dependent amplitude that decays following the inverse-square law. The average call signal received by agent $n$ is denoted by $\overline{\sigma }_{n} \left( t \right)$. In default parameter settings, call ranges are capped at a maximum distance of 125 km but maximum radii of 0 to 500 km are explored. The sign of $\overline{\sigma }_{n} \left( t \right)$ reflects the foraging behaviors of neighboring calling individuals and magnitude integrates the distance to calling whales with the frequency of foraging behaviors.

### State transition matrix and southward migration decisions

Figure 1C defines the possible transitions between the four behavioral states and transition probabilities are dictated by elements of the state transition matrix τ:

$$\tau =\left(\begin{array}{cc}\begin{array}{cc}{S}_{1}\to {S}_{1}& {S}_{1}\to {S}_{2}\\ {S}_{2}\to {S}_{1}& {S}_{2}\to {S}_{2}\end{array}& \begin{array}{cc}{S}_{1}\to {S}_{3}& {S}_{1}\to {S}_{4}\\ {S}_{2}\to {S}_{3}& {S}_{2}\to {S}_{4}\end{array}\\ \begin{array}{cc}{S}_{3}\to {S}_{1}& {S}_{3}\to {S}_{2}\\ {S}_{4}\to {S}_{1}& {S}_{4}\to {S}_{2}\end{array}& \begin{array}{cc}{S}_{3}\to {S}_{3}& {S}_{3}\to {S}_{4}\\ {S}_{4}\to {S}_{3}& {S}_{4}\to {S}_{4}\end{array}\end{array}\right)= \left(\begin{array}{cc}\begin{array}{cc}{p}_{1}& {p}_{2}\\ {p}_{1}& {p}_{2}\end{array}& \begin{array}{cc}{p}^{*}& 0\\ {p}^{*}& 0\end{array}\\ \begin{array}{cc}0& 0\\ 0& 0\end{array}& \begin{array}{cc}{p}_{3}& {p}_{4}\\ {p}_{3}& {p}_{4}\end{array}\end{array}\right),$$

$${p}_{\text{1,2}}={\mathbb{P}}\left({s}_{n}\left(t+1\right)=\left\{\text{1,2}\right\}\right|{s}_{n}(t)=\{\text{1,2}\}), {p}_{\text{3,4}}={\mathbb{P}}\left({s}_{n}\left(t+1\right)=\left\{\text{3,4}\right\}\right|{s}_{n}(t)=\{\text{3,4}\}).$$

Zero elements of the state transition matrix represent unallowed transitions and ${s}_{n}(t)$ indicates the agent’s behavioral state. Southward migration decisions are incorporated into the model via the state transition matrix, specifically in the elements denoted by ${p}^{*}$ that govern the transition probabilities from *S*_1,2_ to *S*_3_. Each of the tested southward migration strategies has a distinct southward transition probability ${p}^{*}$, which are defined in Eqs. (1) and (2) and the Supplement. The remaining transition probabilities are dependent on SST and krill values; these functions are given in the Supplement.

We test a suite of migration strategies, based on a combination of agents personal average foraging effectiveness ${\overline{\omega }}_{n}(t)$ and average received social information $\overline{\sigma }_{n} \left( t \right)$. Foraging effectiveness ${\overline{\omega }}_{n}(t)$ is defined as the average krill intake over a period of *T*-timesteps and gives a time-dependent measure of an individual’s personal foraging success. Foraging effectiveness and social information are averaged to allow individuals to explore their local environment and receive calls from multiple agents.

The following migration strategies prescribe different transition functions for the southward transition probability ${p}^{*}$ based on ${\overline{\omega }}_{n}(t)$ and $\overline{\sigma }_{n} \left( t \right)$.

1. Individual foraging efficiency (personal, *m*_per_ (Table 1))
  1 $${p}_{\text{per}}^{*}={\mathbb{P}}\left({s}_{n}\left(t+1\right)=3|{s}_{n}\left(t\right)\in \{\text{1,2}\}; \overline{{\omega }_{n}}\left(t\right)\right)={\left[1+{\text{exp}}\left({c}_{1}\left(\overline{{\omega }_{n}}\left(t\right)-{c}_{2}\right)\right)\right]}^{-1}.$$
2. Individual foraging efficiency and social communication (personal & social, *m*_per&soc_)
  2 $${p}_{per \& soc}^{*}= {p}_{per}^{*}\cdot {p}_{soc}^{*},$$
  $${\text{where}}\;p_{soc}^{*} = {\mathbb{P}}{(}s_{n} \left( {t + 1} \right) = 3{|}s_{n} \left( t \right) \in \left\{ {1,2} \right\};\;\overline{\sigma }_{n} \left( t \right){ }) = \left[ {1 + {\text{exp}}\left( {c_{3} \left( {\overline{\sigma }_{n} \left( t \right) - c_{4} } \right)} \right)} \right]^{ - 1} .$$

Values of constants are given in Supplementary Table 3 and example transition probabilities are plotted in Supplementary Fig. 3. The personal strategy represents a mechanism based solely on an agent’s foraging efficiency $\overline{{\omega }_{\text{n}}}\left(\text{t}\right)$, or recent average krill intake. The probability ${\text{p}}_{\text{per}}^{*}$ is defined to be high (low) for low (high) foraging efficiencies, meaning that a low foraging efficiency $\overline{{\omega }_{\text{n}}}\left(\text{t}\right)$ increases the chance that agents in the *m*_per_ strategy switch into state ${S}_{3}$ and initiate southward migration. The multiplicative transition probability for the personal and social strategy reflects a strategy where agents initiate migration when both personal and social factors indicate poor foraging opportunities. The probability ${p}_{soc}^{*}$ is defined so agents have a high probability of initiating southward migration if recent received social calls $\overline{\sigma }_{n} \left( t \right)$ indicate low foraging opportunities. Our study focuses on migration strategies centered around an individual’s personal foraging experiences and received social calls. A minimum krill intake requirement was explored as an additional asocial and energetically driven migration mechanism. The minimum krill intake largely did not influence the main outcomes; summary and discussion are included in the Supplement.

### Null model

The results of the migratory models are tested against a hypothetical non-migratory null population (${m}_{0}$, Table 1). These agents follow the two-state transit-forage model of Ref.^56^ and do not receive any southward migration cues. While the non-migratory population is ecologically unrealistic, its output provides a yearly baseline for foraging patterns and krill intake over the modeled foraging season. Transition probabilities between *S*_1_ and *S*_2_ are identical across all presented models, thus the null non-migratory agents receive the same foraging and movement cues as the southward migration models. A yearday-driven migration strategy was also tested as an extrinsically based null model; results can be found in the Supplement.

### Population comparison metrics

Population-level behaviors of each model are compared against empirical data of migration timings and the null model. First, southward migration initiation dates from various migration strategies tested with the IBM are directly compared with data recorded from the Monterey Bay hydrophone. Foraging season durations are known to be latitude dependent^57^, hence hydrophone recordings are compared to the subset of modeled agents whose migration initiated north of Monterey Bay (north of 36° N). To be consistent with the hydrophone recordings, the comparison is with the dates that modeled agents first switched into the southward migration state ${S}_{3}$. Throughout, we refer to the date of southward migration initiation simply as ‘migration date’. A Mann–Whitney U-test^58^ is applied with a null hypothesis that the median migration dates for a modeled strategy have the same distribution as the annual median empirical migration dates determined via hydrophone recordings. The alternative hypothesis is that the model migration dates are from a distribution with earlier migration times.

Second, model migration statistics are compared with published summaries of tagging data from Irvine et al*.*^50^. Prior analysis of 48 blue whale tags spanning 9 years revealed that blue whales exit the U.S. Exclusive Economic Zone (EEZ) in late October, with a mean departure date of October 21^50^. Yearly median EEZ departure dates range from late-September through November and individual whales were recorded departing from late-July through early-January^50^. We compare trends in departure dates for all modelled migrating agents with the reported EEZ departure tagging statistics. We refer to the date that a modeled migrating agent departs from the southern border of the simulation domain simply as ‘departure date’ and specify EEZ departure date to reference the data from empirical tagging statistics^50^. We do not conduct explicit statistical tests between the IBM and empirical EEZ median annual departure dates due to the small number of empirical migrations from tag data when separated by year.

Overall, modelled migration mechanisms are characterized as realistic if the resultant median southward migration and departure dates align with empirical measurements^41,50^ in terms of the correct date range (Oct–Nov) and an interannual flexibility on the order of months.

An agent’s total annual krill intake is defined as the sum of the krill densities at all foraging locations. As a second metric, we compare a population’s total krill intake against that of the hypothetical non-migratory population (*m*_0_). Prey levels are dynamic across years and *m*_0_ acts as a null model representing the maximum yearly krill intake the population could achieve if agents did not migrate and made full use of all the available resources. To compare results across years, yearly krill intakes for each population are represented as a percentage of the median intake of *m*_0_.

To understand the flexibility of migration strategies, performance metrics are analyzed when separated by yearly environmental conditions. The median total krill intake of ${m}_{0}$ is used to categorize each year as low, average, or high krill availability (Supplementary Table 4).

### Parameterization, sensitivity analysis, model robustness

All simulations are initialized with 2000 agents to mimic the population of Eastern North Pacific blue whales. IBM outputs are naturally stochastic and each simulation, even with a fixed parameter set, represents one possible model outcome or realization. To account for the inherent stochasticity and ensure that results are representative of the full range of possibilities, at least 100 simulations are run for each year in 1990–2010 and outcomes report the aggregation of all simulations.

Models were parameterized through a comparison of the simulated migration statistics for years 2000–2010 with the empirical hydrophone migration timings and EEZ departure dates. Robustness in the timings of the migration mechanisms was checked using random parameter samples. Following the Latin Hypercube Sampling procedure^59^, a total of 1000 trials were run for each year in 2000–2010 with parameters randomly selected from a set of realistic, but large intervals. Southward migration dates from the random parameter simulations were compared with those of the empirical data sets. Additional details are included in the Supplement.

## Results

**Figure 2:**
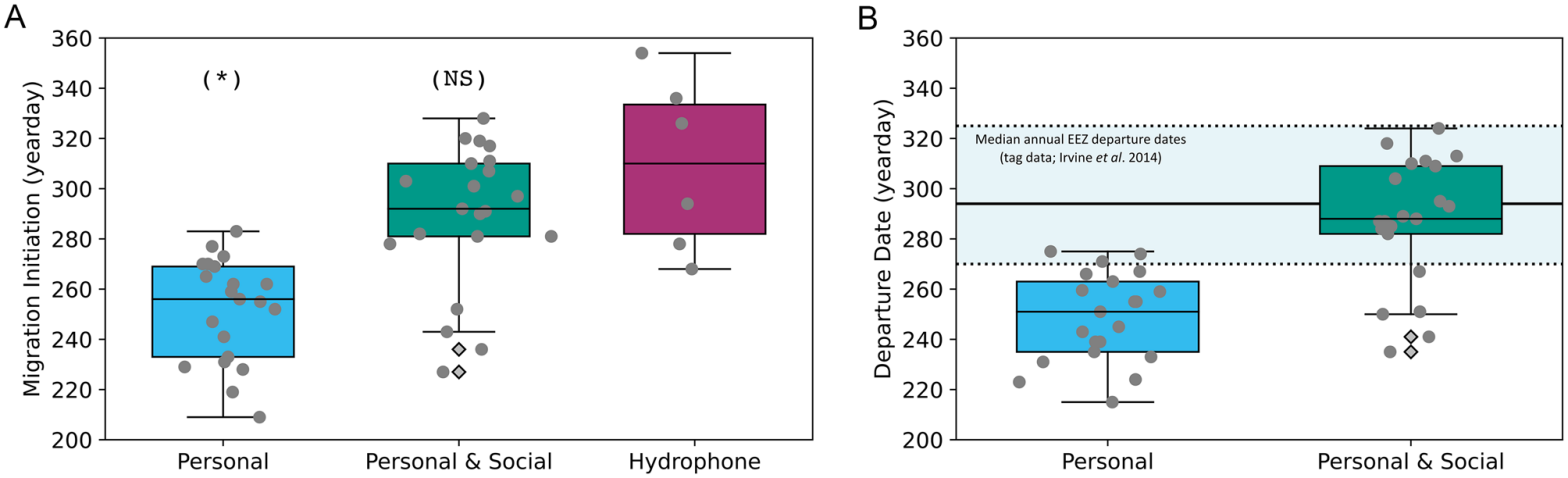
Comparison between modeled and empirical migration statistics. (**A**) Boxplots show the distribution of annual median migration dates (individual years shown in gray dots) for modeled populations (*m*_*per*_, blue; *m*_*per* + soc_, green) and population-level empirical observations (hydrophone dataset^41^). Migration statistics for each modeled migration mechanism were calculated using the subset of the agents whose migration initiated north of Monterey Bay. The (*) label indicates a statistically significant difference between the set of median migration dates of the modeled mechanism and the hydrophone dataset^41^; the (NS) label indicates no significant difference. (**B**) Boxplots show the distribution of median yearly departure dates for modeled populations (*m*_*per*_, blue; *m*_*per* + soc_, green). Median dates for each of these modeled populations are computed yearly for the full migrating population. The thick black line indicates the reported median EEZ departure date of October 21 from empirical, individual-level tag data^50^. The blue shaded region between dashed black lines indicates the extent of median yearly EEZ departure dates in this empirical individual-level data; extent is estimated from data in Ref.^50^.

Southward migration decisions based solely on individual cues (personal information; *m*_per_) yield unrealistically early migrations (Fig. 2A). Median migration initiation dates of *m*_per_ span yearday 209 (late-July) through yearday 283 (mid-October), with a median migration date prior to October 1 in 19 out of 21 years. The median migration dates of *m*_per_ are significantly different from those of the empirical dataset (Mann–Whitney U-test p-value < 0.001; Supplementary Table 5). The departure dates of the *m*_per_ population (Fig. 2B) display similar unrealistically early migration timings that do not overlap with the EEZ departure dates from tag data^50^.

Realistic, late-season migrations that overlap with empirically observed migration timing emerge when social communication is added to the decision process. For the 21 simulated years, the median migration dates of *m*_per&soc_ span late August (yearday 227) through early November (yearday 328). Median migration dates of *m*_per&soc_ fall after yearday 280 (early October) in 75% of the simulated years and are statistically indistinguishable from the timing of southward migration empirically observed from the Monterey Bay hydrophone (p = 0.16; Fig. 2A, Supplementary Table 5). Likewise, the addition of social communication leads to substantial overlap in the modeled and empirical EEZ departure dates (Fig. 2B). Further, a sensitivity analysis revealed that these trends in migration timings for both *m*_per_ and *m*_per&soc_ strategies are robust across a wide range of model parameters (Supplementary Fig. 4).

**Figure 3:**
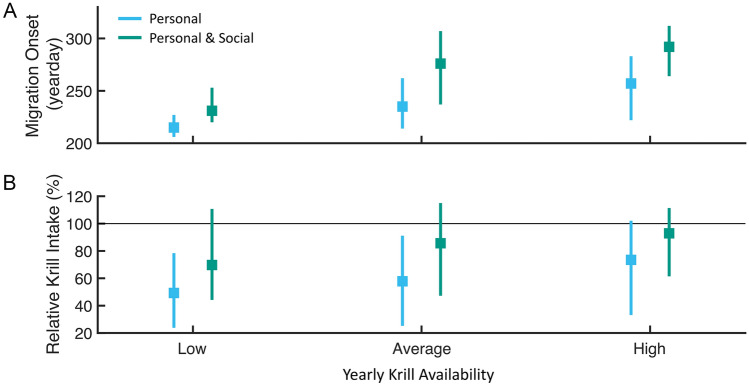
Southward migration distributions and krill intake for modeled populations separated by annual krill availability (low, average, high). Boxplots show median and IQR of (**A**) migration distributions and (**B**) relative krill intake for each modeled migration mechanism. Values in (**B**) are computed as a percentage of the total non-migratory (null) population intake. Grey line indicates the median intake of the null population. Results are aggregated by yearly krill availability on the x-axis.

The relative krill intake of each modeled population is compared to the null non-migratory simulations (*m*_0_) in Fig. 3 with years separated by prey availability. Across all prey conditions, populations using social information (*m*_per&soc_) consume a greater total amount of krill than those using the asocial strategy (*m*_per_). In years with low krill availability, *m*_per_ consumes only 49% of the *m*_0_ krill intake compared to the *m*_per&soc_ median of 70% of the available krill. These trends continue into the average and high krill years, with the asocial population consuming a median of 58% (average krill) and 74% (high krill) of the *m*_0_ intake, and with the socially informed population consuming a median of 85% (average krill) and 92% (high krill) (Fig. 3B, Supplementary Fig. 9).

**Figure 4:**
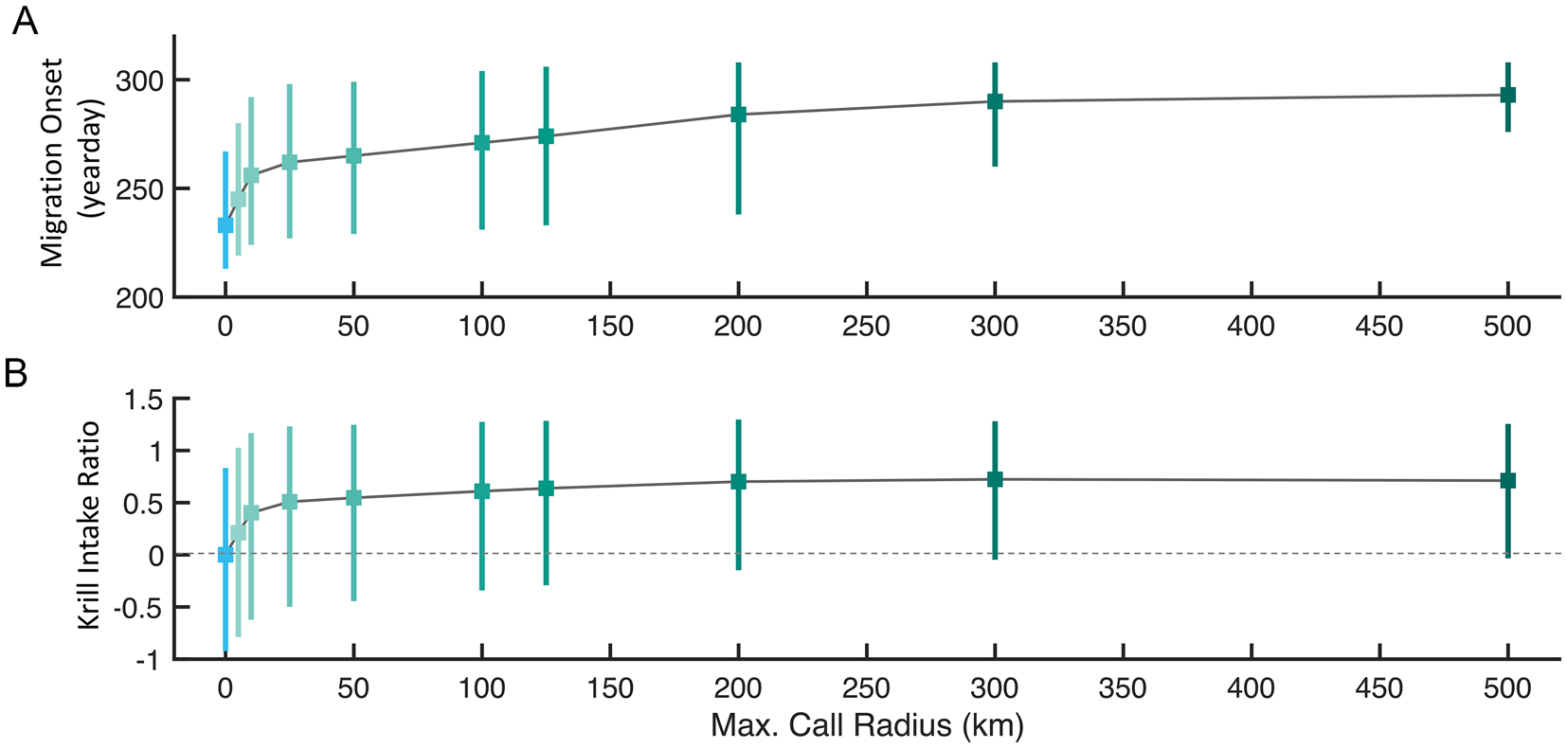
Impact of maximum call radius on modeled migration timing and krill intake. Boxplots show median and IQR of (**A**) migration distributions and (**B**) relative krill intake compared to the personal-only population. Values in (**B**) are computed as a ratio of the deviation from the total personal population ${m}_{per}$ intake. Data aggregated across all 21 years of simulated data. A maximum call radius of 0 km is equivalent to the ${m}_{per}$ population.

The importance of the social communication range was tested by varying the maximum call radius for a range of detection distances (0–500 km, Fig. 4, Supplementary Fig. 10). A maximum call radius of 0 km corresponds to the *m*_per_ strategy (i.e., effectively no social communication). The timing of southward migrations converges to a late season median as the maximum call radius increases (Fig. 4A). Increasing call radius further leads to a decrease in population-wide variation in migration timing, as indicated by the narrowing of the migration timing distribution (Fig. 4A). Likewise, the increase in call radius leads to a krill intake at nearly 175% of the personal-only strategy (Fig. 4B). A relatively small maximum call radius of 5 km leads to a median 12-day delay in departure date and median prey consumption nearly 22% greater than the *m*_per_ krill intake. Maximum call radii of 25 km and above yield at least 50% greater prey intake than *m*_per_ and median migration dates nearly 30 days later.

## Discussion

Understanding the mechanisms of migration is a longstanding question in ecology^24,60,61^. A growing body of research has focused on the role of social interactions in migratory decisions^9–11,16,17^, as social information transfer can enable populations to navigate noisy environmental gradients and can give rise to collective migrations^62^. Yet whether such social cues enable collective migrations and associated fitness benefits at the extreme spatial scale of dynamic pelagic ecosystems has remained untested. Our results indicate that long-distance social interactions are likely an important ingredient in driving collective migration in a long-distance ocean migrant, enabling flexible migration timing and associated benefits in foraging performance.

The recent discovery of an acoustic signature of blue whales’ transition from foraging to migration^42^ prompts the hypotheses that (1) blue whales incorporate long-distance social information from conspecifics in timing their migratory departure from foraging habitat; and (2) this social information improves population-wide foraging outcomes. Using a series of IBMs in conjunction with empirical observations, we find support for these hypotheses, showing that only models including a socially informed strategy enable flexible and realistic migratory departure timing from blue whales’ vast and dynamic foraging habitat (Fig. 2) across a broad range of realistic model parameter values (Supplement). This use of long-distance information from conspecifics enhances population-wide foraging performance under interannual ecosystem variation (Fig. 3).

Optimal foraging theory posits that animals will optimally choose, allocate time to, and depart resource patches in ways that maximize individual fitness^63,64^. However, this model assumes that animals have complete, landscape-wide knowledge of resource availability—an assumption that is typically violated in nature and that is influenced by both patch predictability and individual sensing capabilities. Our results are consistent with theory that predicts the value of nonlocal information to be high in systems with highly transient resource patches, enabling more optimal foraging decisions despite high environmental unpredictability^15^. Many pelagic ecosystems—including blue whales’ foraging habitat in the Northeast Pacific^65^—display these resource dynamics^28–32^, suggesting that nonlocal information should be particularly valuable in this setting. Moreover, our study indicates that theoretical predictions about the utility of social information in heterogenous ecosystems extend to the extreme spatial scale and dynamism of the pelagic ocean. Modeled individuals considering only personal information depart when they experience poor forage conditions locally, even if high-quality foraging opportunities remain elsewhere. This lack of awareness of broader forage availability leads asocial individuals to migrate earlier than empirical observations show (Fig. 2) and results in poorer foraging performance relative to social migrants (Fig. 3B). Delayed migration is enabled by an acoustic social network informing the population about broader forage availability than can be sensed individually (Fig. 3B), thus enabling more optimal foraging and migration decisions. The shortcomings of asocial strategies and success of socially informed migratory decisions are robust to model variation and parameterization (Supplementary Fig. 4). Hence, models including socially informed migratory decisions can consistently better explain trends seen in empirical data relative to asocial-only models, suggesting that long-distance social information is likely an important and necessary source of information in blue whales’ decision of when to depart from foraging habitat and initiate breeding migration.

Social information enhances the range of nonlocal information, but the information acquired personally by information-sharing individuals is often highly correlated^66^. Such homogeneous personal information among individuals can limit the value of social information and the ability of groups to collectively sense broad-scale ecosystem gradients or changes^25^. In pelagic ecosystems where ephemeral resource patches display heterogeneity across a broad range of spatial scales^28,31^, effective collective sensing of broad-scale ecosystem dynamics therefore would require long-distance information transfer between individuals. Blue whales have evolved to take advantage of the potential for long-distance acoustic communication in the aqueous medium of pelagic ecosystems^67^, producing extremely loud and low frequency songs which are detectable over at least tens (if not hundreds) of kilometers^42^. These physical attributes of blue whales’ songs allow for the long-distance communication necessary for collective sensing and migration in the pelagic ocean. While the precise maximum communication range between blue whales is not known, implementing even relatively conservative estimates results in realistic, delayed migration timing and enhanced foraging performance relative to a hypothetical asocial population (Fig. 4). As the social communication radius grows and population-wide migration timing becomes increasingly collective (decreasing IQR in Fig. 4A), the marginal value of social information from increasingly distant conspecifics for foraging performance approaches an asymptote (Fig. 4B). This pattern likely arises from information saturation, where increasingly distant calls provide limited additional information about foraging conditions. As a result, individuals accrue the majority of foraging benefits from social information transfer at moderate ranges of vocal communication (< 50 km; Fig. 4) relative to the detection range of these signals above background noise (at least from human listening devices^42^).

These findings also highlight how the sources of information driving migratory decisions vary depending on the relative value of distinct information sources during different phases of an annual migratory cycle. As Eastern North Pacific blue whales migrate northward in the spring and early summer, individuals have limited personal information about contemporaneous forage conditions, leading to a reliance on spatial memory of long-term foraging hotspots^13^. Moreover, social information transfer via song is sparse during the early foraging season with individuals producing little song during the summer months^41,42,44,68^. As the foraging season progresses and krill prey become less abundant^69,70^, the relative value of long-distance social information should increase as individuals must determine if local prey depletion is representative of broader forage availability. Indeed, song production increases throughout the foraging season, reaching a peak in the late fall when this population begins southward migration on average^42^.

While this social information transfer provides clear benefits to receivers (Fig. 3), the benefits to signalers are not immediately clear. In this situation, one might expect “cheating” to evolve unless song production provides some other fitness benefit to signalers. Song in baleen whales is widely associated with reproduction as it is believed to be a male-specific behavior and peaks on foraging grounds immediately preceding the breeding season^43^. Even if the primary purpose of song relates to reproduction, the diel patterns of blue whale song create secondary information on behavioral state due to diel partitioning of feeding and singing behavior during the foraging state^42^. In this way, the information on behavioral state encoded in diel patterns of blue whale song provides unintentional social information about forage availability to eavesdropping conspecifics. The use of such public information is widespread in animals’ behavioral decisions^20,71^, and its inadvertent nature allows for evolutionary stability even without benefit to the signaler^14^.

Elucidating how animals use various information sources in their migrations advances our understanding of movement ecology in theory and in practice. Discovery of blue whales’ probable use of long-distance social information improves our understanding of this endangered population’s ability to adapt to changing ecosystem conditions in the Anthropocene. Blue whales’ reliance on memory during the northward migration toward foraging grounds suggests limited capacity for this population to adapt to rapid environmental change in this portion of their annual cycle^13^. Yet the ability to flexibly track interannual variation in ecosystem phenology when departing on migration from foraging grounds suggests greater ability to adapt to environmental change^41^. Our findings suggest that this adaptive flexibility is underpinned by long-distance communication—as a result, the already-detrimental impacts of noise pollution on this population might also impair the use of social information in migration timing and its associated foraging benefits^72^. These noise pollution impacts could be particularly detrimental to this endangered population, given that intensive foraging on the summer-fall feeding grounds provides the primary source of energy reserves for fueling their round-trip migration to and from the breeding grounds, as well as rearing of young^35^. Northeast Pacific blue whales’ foraging habitat is home to numerous other species which depend on acoustic communication for various behaviors, and the potential impacts of such noise pollution on these species and behaviors also warrants additional future research and management action. Growing calls and proposed solutions for reducing human impacts on marine soundscapes^72–75^ hold promise for mitigating this impact, aiding in the conservation of marine megafauna and collective migrations. Finally, our results point to a growing need to understand the function and consequences of long-range social communication in migratory populations navigating rapidly changing ecosystems.

### Supplementary Information

Supplementary Information.

## Data Availability

Environmental data from ROMS-NEMUCSC is available at 10.7291/D1KD4J.

## Code availability

Code for simulating and processing the individual-based model is available at 10.5281/zenodo.8305222.
