# Supplementary material for: Long-distance communication can enable collective migration in a dynamic seascape
Source: Sci Rep. 2024 Jun 27;14:14857. doi: 10.1038/s41598-024-65827-2 (PMC11211507; doi:10.1038/s41598-024-65827-2)
Supplement: Supplementary file 1 — Supplementary Information. [file 41598_2024_65827_MOESM1_ESM.pdf]

Supplementary Information: Long-distance communication can enable collective migration in a dynamic seascape

1 Overview, Design concepts, and Details (ODD)

This section of the supplementary information is formatted under the Overview, Design concepts, and Details (ODD) protocol (Grimm et al., 2006; Grimm et al., 2010; Grimm et al., 2020).

1.1 Purpose

The purpose of this individual based model (IBM) is to investigate the role of foraging and long-range social information in driving the yearly southward breeding migration of Northeastern Pacific blue whales.

1.2 Entities, state variables, and scales

Entities or agents in the IBM represent a single whale. State-variables track how each agent moves through and experiences the domain. The domain spans 116-128°W and 32-44°N and is divided into 3 km × 3 km spatial patches. Each patch is assigned a sea surface temperature (SST; °C) and near surface krill abundance. The SST and krill density are updated daily (24 hours) and are provided from an implementation of ROMS (see 1.6).

To investigate drivers of southward migration, we use a series of IBMs, each of which is designed with a distinct southward migration mechanism based on a combination of environmental and social information. The IBMs are formulated as a state-switching model with four behavioral states representing transit and forage behaviors during the foraging season and breeding migration. In all models, each agent is assigned the following state-variables: behavioral state, location, SST, and krill (Table 1). In models with social calls the three additional state variables of sex, calling behavior, and received call signals are assigned.

One time step represents 6 hours (4 time steps/day) and simulations were run for 180 days. Each simulations includes 2,000 agents. Simulations are run for years 1990-2010.

1.3 Process overview and scheduling

The model progresses in 6 hour time steps. Within each time step, the state-variables for each agent are updated following the order displayed in Figure 1. Environmental state variables are updated every 24 hours

Supplementary Table S 1: Description of state-variables defined to each agent. Variables are unitless, unless otherwise specified. An asterisk next to the state variable name indicates a variable that is only used in models with social calls.

**State Variable**	**Description**
Behavioral state	Value of 1-4 defining transiting and foraging behavior
Location	(x, y)-coordinate pairs, giving the distance (in meters) from the southwest corner of the domain. Continuous variable.
krill	Value of ROMS krill at location of agent on each time step
SST	Temperature value (°C; ROMS) at location on each time step
*Sex	Assigned male or female at random, time independent
*Calling behavior	Value of 0 or 1 indicates if agent is calling at current time step
*Received call signals	Average value of calls heard at each time step

(4 time steps). Details of each process are included in Section 2. Movement updates are selected from state-dependent step length and turning angle distributions.

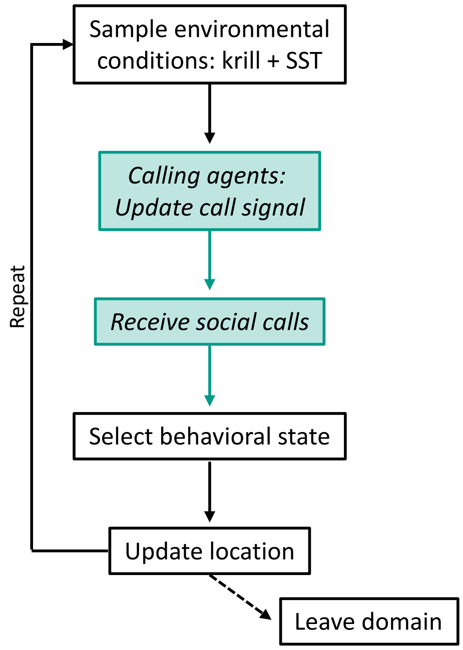

Supplementary Figure S 1: Schematic of underlying IBM algorithm. The full process represents a single time step and is completed for each agent. The components in teal boxes are only relevant for models with social calls.

1.4 Design concepts

Basic Principles: The presented IBM takes the form of a state-switching model, with behavioral states capturing the distinct movements associated with transiting, foraging, and migrating behaviors. Behavioral

states and their associated movement distributions are based on tagging data (Bailey et al., 2009) and have been successfully incorporated into IBMs (Dodson et al., 2020).

In this study, multiple southward migration mechanisms based on environmental and social cues are tested via the state transition functions that control how agents enter the migratory states. Migration mechanisms are evaluated by comparing the timing of modeled southward migrations to empirical hydrophone observations (Oestreich et al., 2022; Oestreich et al., 2020). Thus, the model will provide insights about the processes underlying blue whale migrations.

Emergence: The existence and timing of the southward breeding migration is an emergent behavior that arises from foraging behaviors and received social calls. The emergent southward migration is the main output of interest. Additional emergent population-level behaviors include seasonal foraging trends and locations.

Adaptation: Agents update their behavioral state on each time step based on the environmental conditions and received social information. Behavioral states dictate the step length and turning angle of the next movement update, mimicking movements associated with transit and forage behaviors (Bailey et al., 2009).

Objectives: There are no model processes that seek to maximize some objective.

Learning: Agents do not learn throughout the model implementation.

Prediction: Agents do not make any predictions of future consequences of their decisions.

Sensing: It is assumed that agents can locally sense the environmental state variables. Agents are able to sense the SST and krill values of neighboring grid cells and can determine the direction of highest foraging probability (if one exists).

Agents can sense and receive social calls within a maximum radius of their location. The default radius is set at 125 km. Agents are assumed to be able to sense ocean versus land. Movement updates that bring agents onto land are not allowed.

Interaction: Agents interact through social calls. Calls include information about the call sender's recent foraging behaviors (see Section 2). The information shared impacts southward migration decisions. In models without social communication, agents do not interact.

Stochasticity: The selection of behavioral states and movement updates is inherently stochastic. Behavioral states are selected probabilistically given a state transition matrix. Movement updates are selected randomly from associated turning angle and step length distributions. Stochasticity is used to reproduce variability in the modeled decisions and outcomes.

Collectives: Agents do not belong to any collective unit.

Observation: During model simulations, the behavioral state, cumulative krill intake, average recent krill intake, calling behavior, and received calls are recorded for each agent on each time step. These quantities contribute to the selection of behavioral states and provide a useful comparison between distinct migration mechanisms.

Calling behavior only impacts the behavioral states and decisions of agents in the models with social behavior. However, in practice, calling behavior can be monitored in all models.

1.5 Initialization

Since we are interested in factors driving the breeding migration, models are initiated on July 1st. Agents' initial locations are selected uniformly at random within areas of climatologically high krill densities from the ROMS data Abrahms et al., 2019. Regions of climatologically high krill were defined by averaging the krill abundance on July 1st 1990-2010 for each spatial grid cell, then thresholding the averages.

At the start, 50% of agents are randomly assigned to be male, an important fact since the primary producer of long-distance song are males.

1.6 Input data

The environmental variables SST and near-surface krill abundance are precomputed and supplied as input data to the IBM. These variables are precomputed using a Regional Ocean Modeling System (ROMS) implementation that is specifically parameterized for the California Current region (Fiechter et al. 2018; Fiechter et al. 2020). Additionally, the ROMS implementation has been coupled with NEMUCSC, a biogeochemical model adapted from the North Pacific Ecosystem Model for Understanding Regional Oceanography of Kishi et al. (2007), and provides the krill concentrations. ROMS-NEMUCSC data that spans the domain is available for the years 1990-2010 at 3km spatial resolution and daily temporal resolution. Simulated krill concentrations have been evaluated against existing in situ data for May-June and are expected to adequately reproduce observed krill aggregation regions during the upwelling season (Fiechter et al., 2020). This data is available at https://doi.org/10.7291/D1KD4J.

2 Submodels and Model Details

This section represents the *Submodels* portion of the ODD protocol. Subsections define the processes in Figure 1.

Throughout the model description, the subscript n will indicate a quantity or variable for an individual agent, where $n \in \{1, 2, \ldots, 2000\}$. Additionally, t is used to represent the time step of the simulation. Model time steps are 6-hours in length and simulations are initiated on July 1 (t_0). The yearday $\tilde{t}$ can be extracted from the time step t using the floor-function as $\tilde{t} = t_0 + \lfloor \frac{t}{4} \rfloor$. Variables are summarized in Table 2 and all notation and variable names are consistent with that of the main text. Additionally, variables with an overbar (for example, $\bar{x}_n$) are consistently defined to be averages in time.

2.1 Social Communication

The social communication process is divided into the call producers and call receivers.

2.1.1 Call Producers

The call signal sent by agent n on at time step t is defined as

$$\delta_n(t) = \tanh\left[b_1\left(\bar{x}_n(t) - b_2\right)\right]$$

where $\bar{x}_n(t)$ denotes the average 24-hour foraging behavior

$$\bar{x}_n(t) = \frac{1}{4}\sum_{j=1}^{4} \mathbb{1}_{2,4}\left(s_n(t-j)\right), \qquad \mathbb{1}_{2,4}\left(s_n(t)\right) = \begin{cases} 1 & s_n(t) \in \{2,4\} \\ 0 & s_n(t) \in \{1,3\} \end{cases}.$$

Here, $\bar{x}_n(t)$ takes on a value between 0 and 1, with 0 representing all transit and 1 all forage behaviors. The call signal $\delta_n(t)$ is intended to be a time-dependent measure of foraging success analogous to the ratio $\mathrm{CI}_{\mathrm{night}}$:$\mathrm{CI}_{\mathrm{day}}$ (Oestreich et al., 2022; Oestreich et al., 2020).

At the start of the simulation, 50% of agents are randomly assigned as male (call producers) and on each time step, a subset of male whales are randomly selected to produce calls. The proportion of male whales calling on each time step increases linearly from 5% on July 1 to 30% on December 31.

2.1.2 Call Receivers

Define $M_n(t)$ to be the subset of whales whose calls are heard by whale n at t, $N_n(t) = |M_n(t)|$ (cardinality of the set $M_n(t)$) to be the number of calls received, and $R_{j,n}(t)$ the distance (in meters) between two agents n and j. Then, the call signal received by whale n at time t, $\sigma_n(t)$, is an average of all calls received on that timestep, weighted by the inverse-square law dependent decay of the call amplitude

$$\sigma_n(t) = \frac{1}{N_n(t)} \sum_{j \in M_n(t)} ISL_j(t)\delta_j(t), \qquad ISL_j(t) = \begin{cases} \left[A_0 - \left|20\log_{10}\left(\frac{1}{R_{j,n}(t)}\right)\right|\right] & R_{j,n}(t) < R_{\max} \\ 0 & R_{j,n}(t) \geq R_{\max} \end{cases}.$$

Call signals are capped at a maximum radius $R_{\max}$. Calling behaviors and the inverse-square law amplitude decay are independent of call radii.

Variable	Description
$s_n(t)$	Behavioral state of whale n at time step t
$\rho_n(t)$	Krill density at location of whale n at time step t
$\bar{x}_n(t)$	Daily foraging rate of whale n at time step t
$\delta_n(t)$	Call signal sent by whale n at time step t
$\sigma_n(t)$	Call signal received by whale n at time step t
$\bar{\sigma}_n(t)$	Average call signal received by whale n over the past T time steps
$\bar{\omega}_n(t)$	Foraging effectiveness of whale n at time step t (averaged over previous T time steps)
τ	State transition probability matrix

Supplementary Table S 2: **Model variables.** Summary and descriptions of model variables.

Parameter	Description	Default Value	Range of Values
**Calling Behavior**			
b_1	Steepness of call signal	8	[3, 10]
b_2	Call signal threshold	0.38	[0.3, 0.4]
A_0	Strength of call at source	180 dB	
$R_{\max}$	Maximum call radius	125 km	
**Transition to Southward Migration**			
c_1	Steepness of foraging transition probability	15	[10, 20]
c_2	Foraging transition threshold	0.2	[0.05, 0.3]
c_3	Steepness of social call transition probability	0.22	[0.1, 0.25]
c_4	Social call transition threshold	-10	[-20, 0]
$\kappa_{\min}$	Minimum krill intake threshold	100	

Supplementary Table S 3: **Calling and migration model parameters.** Summary and descriptions of model parameters related to calling behavior and southward migration. Model results computed using default parameter values. Sensitivity analysis conducted over the listed range of values. Parameter values with no listed range were not included as part of the sensitivity analysis. Parameters without units are unitless.

2.2 Selecting behavioral states

The four behavioral states $\mathcal{S}_{1,2,3,4}$ represent transiting and foraging behaviors during the northward foraging and southward breeding migrations. Each behavioral state is associated with characteristic movements defined by step length and turning angle distributions.

Transitions between the four behavioral states $\mathcal{S}_{1,2,3,4}$ are governed by the state transition probability matrix τ defined by

$$\begin{aligned}
\tau &= \begin{pmatrix} \mathcal{S}_1 \to \mathcal{S}_1 & \mathcal{S}_1 \to \mathcal{S}_2 & \mathcal{S}_1 \to \mathcal{S}_3 & \mathcal{S}_1 \to \mathcal{S}_4 \\ \mathcal{S}_2 \to \mathcal{S}_1 & \mathcal{S}_2 \to \mathcal{S}_2 & \mathcal{S}_2 \to \mathcal{S}_3 & \mathcal{S}_2 \to \mathcal{S}_4 \\ \mathcal{S}_3 \to \mathcal{S}_1 & \mathcal{S}_3 \to \mathcal{S}_2 & \mathcal{S}_3 \to \mathcal{S}_3 & \mathcal{S}_3 \to \mathcal{S}_4 \\ \mathcal{S}_4 \to \mathcal{S}_1 & \mathcal{S}_4 \to \mathcal{S}_2 & \mathcal{S}_4 \to \mathcal{S}_3 & \mathcal{S}_4 \to \mathcal{S}_4 \end{pmatrix} \\
&= \begin{pmatrix} p_1 & p_2 & p^* & 0 \\ p_1 & p_2 & p^* & 0 \\ 0 & 0 & p_3 & p_4 \\ 0 & 0 & p_3 & p_4 \end{pmatrix}, \qquad
\begin{aligned} p_1 &= \mathbb{P}\left(s_n(t+1) = 1 | s_n(t) \in \{1,2\}\right) \\ p_2 &= \mathbb{P}\left(s_n(t+1) = 2 | s_n(t) \in \{1,2\}\right) \\ p_3 &= \mathbb{P}\left(s_n(t+1) = 3 | s_n(t) \in \{3,4\}\right) \\ p_4 &= \mathbb{P}\left(s_n(t+1) = 1 | s_n(t) \in \{3,4\}\right) \end{aligned}
\end{aligned}$$

where $s_n(t) \in \{1, 2, 3, 4\}$ is the behavioral state of whale n at time step t. Allowed transitions are show in the schematic in Figure 1 in the main text.

Southward migration strategies are incorporated in the southward transition probability p^*. Tested migration strategies are based on a combination of an agent's personal foraging behavior and received social information. As defined in the main text, we set $\bar{\omega}_n(t)$ to be the average foraging effectiveness (average krill intake) and $\bar{\sigma}_n(t)$ to be the average received social information of agent n over a period of $T = 40$ timesteps (10 days). These are defined by

$$\bar{\omega}_n(t) = \frac{1}{T} \sum_{j=1}^{T} \mathbb{1}_{2,4}\left(s_n(t-j)\right) \cdot \rho_n(t-j), \qquad \bar{\sigma}_n(t) = \frac{1}{T} \sum_{j=1}^{T} \sigma_n(t-j).$$

Migration strategies are encoded in the transition probability $p^* = \mathbb{P}\left(s_n(t+1) = 3 | s_n(t) \in \{1,2\}\right)$. The transition probability functions associated with each strategy are given below. Although the subscript n is omitted, all transition probabilities are assigned for each agent. All parameters are included in Table 3.

1. Individual foraging efficiency (personal):

$$p^*_{\text{per}} = \mathbb{P}\left(s_n(t+1) = 3 | s_n(t) \in \{1,2\};\ \bar{\omega}_n(t)\right) = \left[1 + \exp\left(c_1\left(\bar{\omega}_n(t) - c_2\right)\right)\right]^{-1}$$

2. Individual foraging efficiency and minimum krill intake (personal & min krill):

$$p^*_{\text{per \& krill}} = p^*_{\text{per}} \cdot p^*_{\text{krill}}$$

3. Social communication (social):

$$p^*_{\text{soc}} = \mathbb{P}\left(s_n(t+1) = 3 | s_n(t) \in \{1,2\};\ \bar{\sigma}_n(t)\right) = \left[1 + \exp\left(c_3\left(\bar{\sigma}_n(t) - c_4\right)\right)\right]^{-1}$$

4. Individual foraging efficiency and social communication (personal & social):

$$p^*_{\text{per \& soc}} = p^*_{\text{per}} \cdot p^*_{\text{soc}}$$

The personal, social, and personal & social strategies and their results are described in detail in the main text. In the Supplementary Results (Section 8), we additionally include results of a migration strategy with a minimum krill intake requirement which acts as a proxy for an energetically-driven migration strategy. Specifically, agents are only able to migrate if their krill intake exceeds a minimum krill threshold κ_{min}. Thus, define the function

$$p^*_{\text{krill}} = \begin{cases} 1 & \kappa_n(t) > \kappa_{\text{min}} \\ 0 & \kappa_n(t) \leq \kappa_{\text{min}} \end{cases}$$

where the cumulative krill intake $\kappa_n(t)$ is found by summing the krill density ρ at the agent's foraging locations

$$\kappa_n(t) = \sum_{j=1}^{t} \mathbb{1}_{2,4}\left(s_n(t-j)\right) \cdot \rho_n(t-j).$$

State transition probabilities are computed as follows. First, p^* is computed and fixed. Then, for agents in $\mathcal{S}_{1,2}$ the probabilities p_1 and p_2 are defined using the fact that the rows of the STPM sum to 1. Thus, the probabilities are set to

$$p_2 = (1 - p_*) \left[\mathbb{P}_{E,K}\left(s_n(t+1) = 2 | s_n(t) \in \{1,2\}; SST, \rho\right)\right], \qquad p_1 = 1 - p^* - p_2$$

where $\mathbb{P}_{E,K}\left(s_n(t+1) = 2 | s_n(t) \in \{1,2\}; SST, \rho\right)$ is the probability of foraging due to SST and krill density defined in (Dodson et al., 2020). Parameter values for the forage-transit selection process are identical to those in (Dodson et al., 2020). Transition probability functions between $\mathcal{S}_1$ and $\mathcal{S}_2$ are identical across all presented models.

For agents in $\mathcal{S}_{3,4}$, we likewise define the probability of foraging and utilize that $p_3 + p_4 = 1$. Thus,

$$p_4 = \mathbb{P}_{E,K}\left(s_n(t+1) = 4 | s_n(t) \in \{3,4\}; SST, \rho\right)$$
$$p_3 = 1 - p_4$$

where p_4 has a stricter foraging threshold (high krill density required for foraging). Transition probabilities between $\mathcal{S}_3$ and $\mathcal{S}_4$ are identical across all presented models that include the southward behavioral states.

2.2.1 Sampling Environmental Conditions and Movement Updates

On each time step, agents sample and store as state variables the SST and krill density at their current location (values from simulated ROMS-NEMUCSC data). These environmental parameters are used to compute the elements of the state transition probability matrix.

Regardless of migration strategy, movement updates are selected from distinct turning angle and step length distributions associated with characteristic transit and forage behaviors. That is, movement updates depend on the selected behavioral state. These movement distributions were parameterized from empirical data (Bailey et al., 2009) and are additionally defined in previous model iterations (Dodson et al., 2020). We point to these sources for further details.

Turning angles for states $\mathcal{S}_{1,2,4}$ are defined as a deviation from the current trajectory of an individual, with a 0° turning angle corresponding to an agent continuing straight along its path connecting the locations at the current and previous time steps. For agents in state $\mathcal{S}_3$, a 0° turning angle corresponds to southward migration with the agent heading towards latitude 32°N and longitude 119°W; this point was chosen to replicate a southward migration trajectory consistent with heading toward the southern breeding grounds.

3 Model Assumptions

Here, we summarize and clarify model assumptions.

- Behavioral states and positions are updated every six hours. Transition probabilities within the north and southward migration categories (between states $\mathcal{S}_1 \rightarrow \mathcal{S}_2$ and $\mathcal{S}_3 \rightarrow \mathcal{S}_4$) are based only on SST and prey levels.
- Transition probabilities between $\mathcal{S}_1 \rightarrow \mathcal{S}_2$ are identical across all presented models.
- Transition probabilities between $\mathcal{S}_3 \rightarrow \mathcal{S}_4$ are identical across all presented models that include the southward behavioral states.
- Individuals commit to southward migration and are not permitted to transition from states $\mathcal{S}_{3,4}$ to $\mathcal{S}_{1,2}$.
- Agents are not bound to the domain and will freely leave if their movement updates take them outside the domain.
- Since we are interested in factors driving the breeding migration, the model is initiated on July 1st. Agents' initial locations are selected uniformly at random within areas of climatologically high krill densities from the ROMS data (Abrahms et al., 2019).
- Tested migration mechanisms represent strategies based only on foraging history, information gathered from social calls, and year-day (null model).

4 Null Models

Model results are tested against two null models. The first null model, a hypothetical non-migratory population, is described in the main text and is the two-state model of Dodson et al., 2020. The second null model follows a yearday-driven migration strategy. Migration dates for individuals in the yearday model are pre-determined and follow a normal distribution with a mean of 310 (November 6) and standard deviation of 20 days. The mean and standard deviation represent average historical trends in blue whale migrations and average call behavior recorded from the MARS hydrophone (Oestreich et al., 2022; Oestreich et al., 2020). The yearday-driven null model provides a extrinsically-based modeled population for comparison.

5 Software

All simulations of the IBM were programmed in MATLAB and run using MATLAB Version 9.13 (R2022b) (The MathWorks Inc., 2022a). Since each simulation run is independent from others, the simulations were run in parallel using the the parallel computing toolbox (The MathWorks Inc., 2022b). Code for simulating and processing the individual-based model is available at https://doi.org/10.5281/zenodo.8305222.

Statistical analysis using the Mann-Whitney U-test (Mann and Whitney, 1947) was conducted with Python Version 3.10.8 using the `mannwhitneyu` function from the stats module of SciPy (Virtanen et al., 2020).

6 Yearly Krill Availability

The 1990-2010 ROMS-NEMUCSC data was separated into years of low, average, and high krill availability based on the median total krill intake of the null non-migratory population. Years were classified as "average" if the median krill intake fell roughly in the middle 50% of the data. Yearly median krill intakes of the non-migratory population are displayed in Figure 2. The horizontal lines divide years based on the classifications of low, average, and high krill availability.

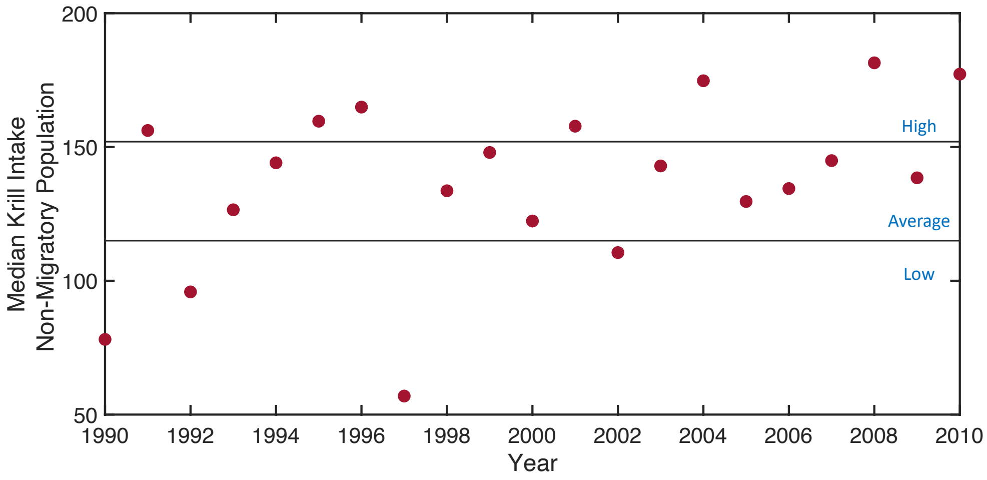

Supplementary Figure S 2: **Non-migratory population median yearly krill intake.** Median yearly krill intake of the null non-migratory population. Horizontal lines divide the years into those classified as low, average, and high krill availability.

7 Sensitivity analysis and model parameterization

Robustness of the timing of the migration mechanism was tested with using random parameter samples. Realistic, but large intervals were set for all parameters (Table 3). A total of 1,000 trials were run for each year in 2000-2010 with parameters randomly selected from the set parameter ranges using Latin Hypercube Sampling procedures (Marino et al., 2008). Figure 4 shows the median migration dates for all random sample trials compared with statistics from empirical tagging (Irvine et al., 2014) and hydrophone (Oestreich et al., 2022) data. Tested parameter intervals were consistent across all strategies.

Parameters c_1, c_2, c_3, c_4 (Table 3) define the transition probabilities to southward migration. Southward transition probability functions take the form of a selection function, with a high (low) probability of southward migration when conditions are poor (rich). The form of these probability transition functions for the tested range of parameters is shown in Figure 3. The range of parameters was selected to capture a broad range of reasonable migration decisions and a large space of functional forms of the transition functions.

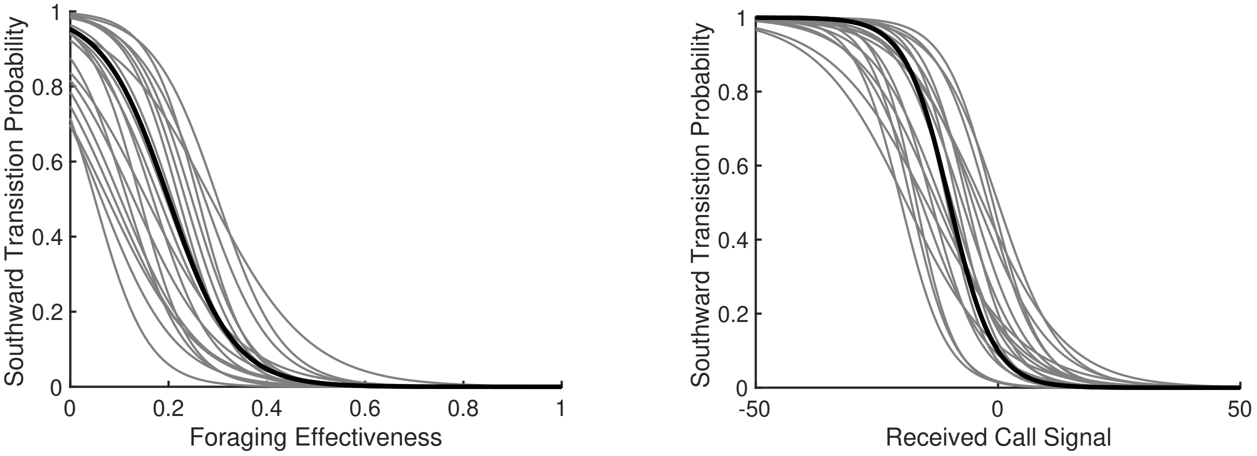

(a) (b)

Supplementary Figure S 3: Southward transition probability functions. Gray curves show 20 example forms of the transition functions for (a) p^*_{per} and (b) p^*_{soc} under the range of tested parameter values c_1, c_2, c_3, c_4. Black curves show the transition function using the default parameter values.

The addition of social calls leads to late season migrations over the full range of realistic parameters. Notably, Figure 4 shows that even the lowest median migrate dates for social strategies are within reason. The asocial strategies consistently lead to early migrations over the broad range of parameters with median dates as early as September - even when restricted to the subset of the population that migrates from the higher latitudes. The results of this sensitivity analysis are important - they show that including social information in migration decisions leads to a late season migration over a broad range of parameters and conditions.

The default set of parameters stated in Table 3 and used for the results in the main text were selected to

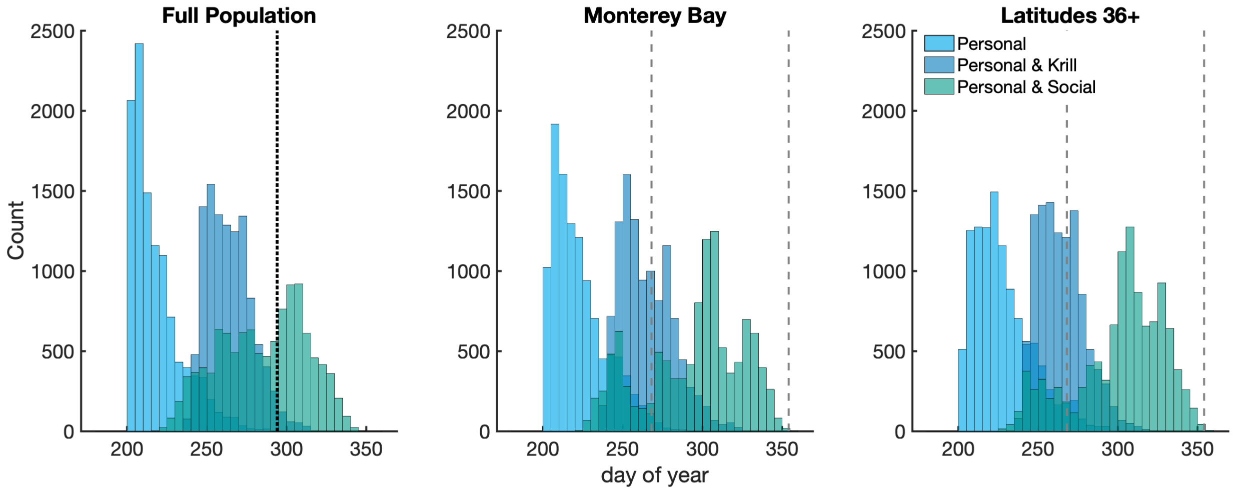

Supplementary Figure S 4: **Histograms of median southward migration dates from random parameter trials.** Data given for (a) the full population, (b) the portion of the population that migrated from latitudes 36-37 (Monterey Bay), and (c) the portion of the population that migrated from latitudes 36+. Veritical dashed lines provide comparisons to empirical data. Black dotted line in (a) indicates the median migration date from tagging studies (Irvine et al., 2014). Gray dashed lines in (b) and (c) indicate the minimum and maximum extent of median migration dates from the Monterey Bay hydrophone (Oestreich et al., 2022).

give later season migrations in the personal-only population (yielded migration timings in the upper tails of the distributions in Figure 4).

8 Supplementary Results & Discussion

8.1 Comparison of Migration Strategies

Here, we include results from the additional null-yearday and minimum krill intake migration strategies. As was done in the main text and described in the section *Population Comparison Metrics*, two comparisons are done between the modeled agents and empirical data. First, yearly median migration dates from the Monterey Bay hydrophone (Oestreich et al., 2020) are compared to the yearly median migration dates of the simulated populations; specifically to the subset of simulated modeled agents whose migration initiated north of Monterey Bay (north of $36°N$). This selection is done to best compare the model data with the empirical results from the Monterey Bay hydrophone, which has a 100 km listening range. Furthermore, migration dates are believed to be latitude-dependent. Simulated agent's migration dates were recorded as the year day that they switched into $\mathcal{S}_3$.

Median migration dates of all strategies are compared to the hydrophone dataset in Figure 5. A Mann-

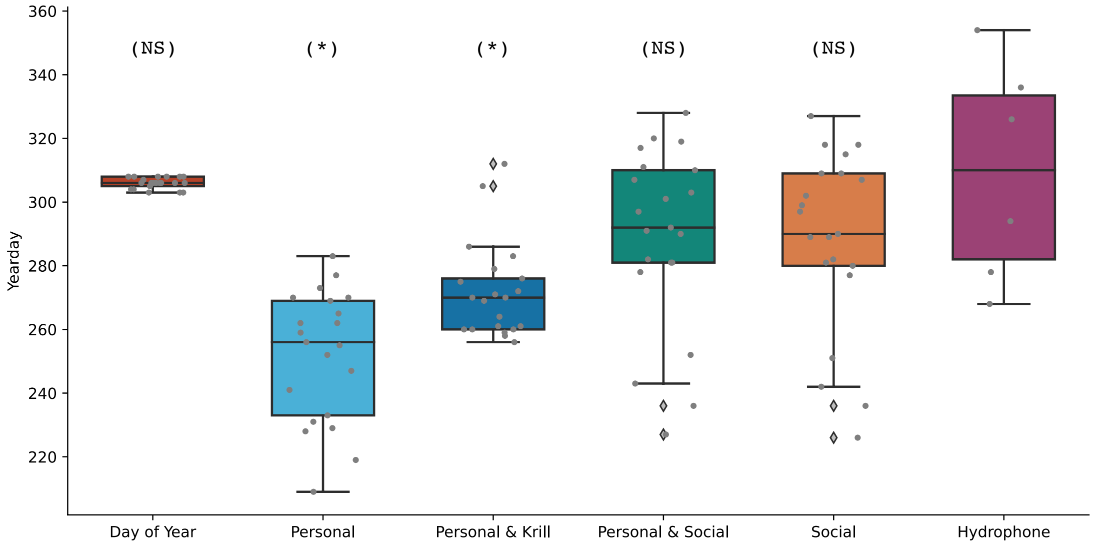

Supplementary Figure S 5: **Comparison of migration dates from Monterey Bay hydrophone data and modeled migration mechanisms.** Model data aggregated across all 21 years. Migration statistics for each modeled migration mechanism were calculated using the subset of the agents whose migration initiated north of Monterey Bay (specifically north of 36°N). Boxplots show the distribution of year median migration dates (shown in gray dots). The boxplot labeled hydrophone is empirical data from the Montery Bay hydrophone Oestreich et al., 2020. The (*) label indicates a statistically significant difference between the set of median migration dates of the modeled mechanism and the hydrophone dataset and the (NS) label indicates no significant difference.

Whitney U-test (p-values in Table 4) indicates that both the set of median migration dates for the personal and personal & minimum krill strategies are statistically different from the set of hydrophone median migration dates. The migrations of the socially informed populations and null day of year are not statistically significantly different from the hydrophone data. However, the spread of the null yearday population is much narrower than the empirical hyrdophone data.

Second, the median migration dates of the full simulated populations (not just the individuals whose migration initiated north of Monterey Bay) are compared to each other and the median date of blue whales departing the U.S. Exclusive Economic Zone (EEZ) found from empirical tagging studies in Irvine et al., 2014. The EEZ departure dates from Irvine et al., 2014 include a large range of whale locations and latitudes, hence it is appropriate to compare this set of empirical data with the full simulated population.

Figure 6 shows the median migration dates of the full population of all strategies compared with the median EEZ departure date. The dashed purple line shows October 21, the median date of blue whales departing the U.S. Exclusive Economic Zone (EEZ) found from empirical tagging studies in Irvine et al., 2014. On average, the migration dates of the full population are lower than the subset whose migration initiated from northern latitudes. Additionally, migration dates of the social migration strategies are found to be compatible with

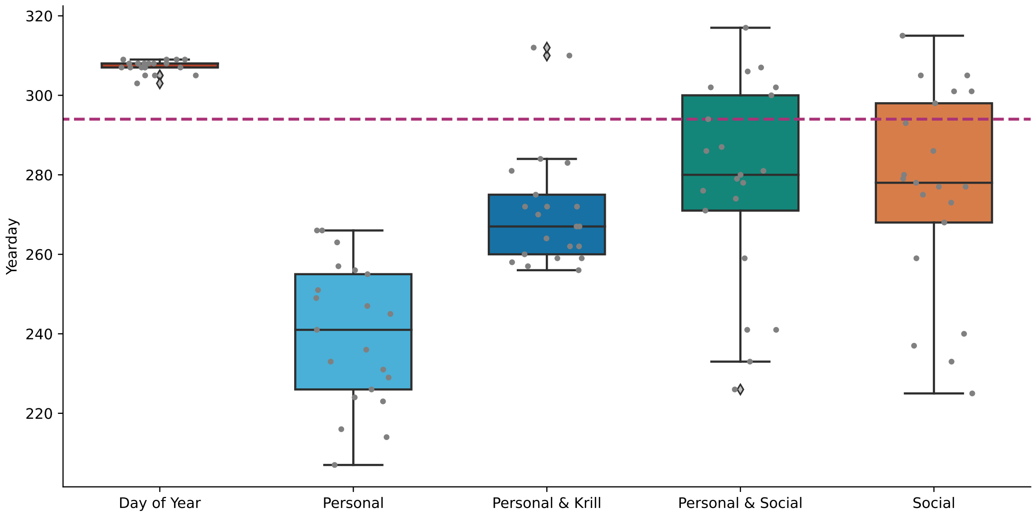

Figure 6: **Comparison of migration dates from empirical tagging data and modeled migration mechanisms.** Model data aggregated across all 21 years. Purple dashed line shows the median EEZ departure date from the empirically tagged population in Irvine et al., 2014.

those from (Irvine et al., 2014).

**Migration Strategy**	**p-value**
Day of Year	0.51
Personal-Only	0.000149 *
Personal & Krill	0.00416 *
Personal & Social	0.16
Social-Only	0.16

Supplementary Table S 4: Results of the Mann-Whitney U-test to determine if distributions of median migration dates are identical to the hydrophone data. The p-values are rounded to 3 significant digits. A * indicates a statistically significant difference with a p-value < 0.005.

Representative yearly migration distributions are shown for the (null) yearday, personal, and personal & social strategies in Figures 7-8. The distributions for the personal & social population consistently show late-season migrations with one or more southward migration waves. Multiple migration waves is consistent with similar patterns observed in hydrophone data (Oestreich et al., 2022).

The migration dates of the null yearday population are largely consistent across yearly prey conditions and the population is unable to respond to the dynamic environment (Figure 9). The yearday population does maintain a high krill intake across the various prey conditions, largely due to the population remaining in the domain for almost the entire foraging season. The minimum krill intake requirement delays the southward migration dates (as compared to the personal population, Figures 5-9). However, the minimum prey intake

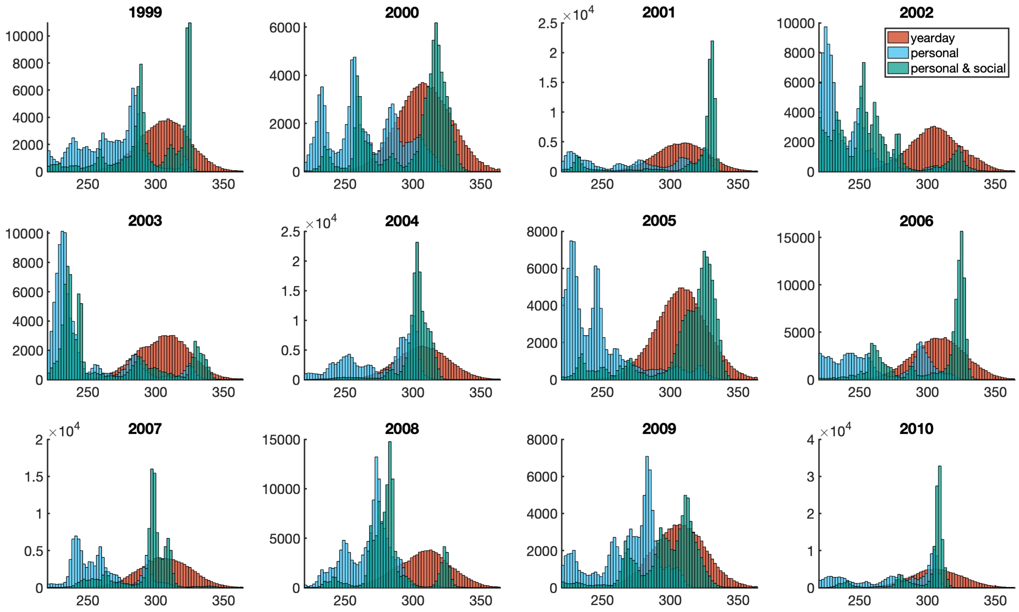

Supplementary Figure S 7: **Yearly migration distributions.** Representative migration distributions of personal, personal & social, and day of year migration strategies. Histograms show the yearday of migration initiation. Subset of population whose migration initiated north of Monterey Bay. Data compiled from 100 simulations for each year, with 2,000 agents per simulation.

also reduces the flexibility and ability to adapt to dynamic prey conditions. In years with lower than average prey, the personal & krill population has later southward migration dates as the agents continue foraging in poor conditions in an attempt to achieve the minimum prey intake.

Finally, here we also provide some comparison between the empirical Monterey Bay hydrophone and an in-silico hydrophone added into the IBM in Monterey Bay. The in-silico hydrophone was added to the IBM to best mimic the recordings of the physical one. The in-silico hydrophone has a maximum listening radius of 100 km and averages all call signals $\delta(t)$ unweighted by distance. Figure 10 displays the in-silico hydrophone data binned by month and aggregated across years 1990-2010 for the personal and personal and social populations. The format of Figure 10 is intended to directly mimic the monthly binned ratio of day versus night call intensities reported by Oestreich *et al.* (2020) (see Figure 2C in Oestreich et al., 2020). Recall that a call signal near one (1) represents an agent who exhibited a high level of foraging behavior over the previous 24 hours and a call signal near negative one (-1) indicates an agent who exhibited a high level of transiting behavior over the previous 24 hours.

The trends in Figure 10 are consistent with those in Figures 5-6. Namely, the population in the personal model exhibits early southward migrations, as demonstrated by the population level switch from foraging

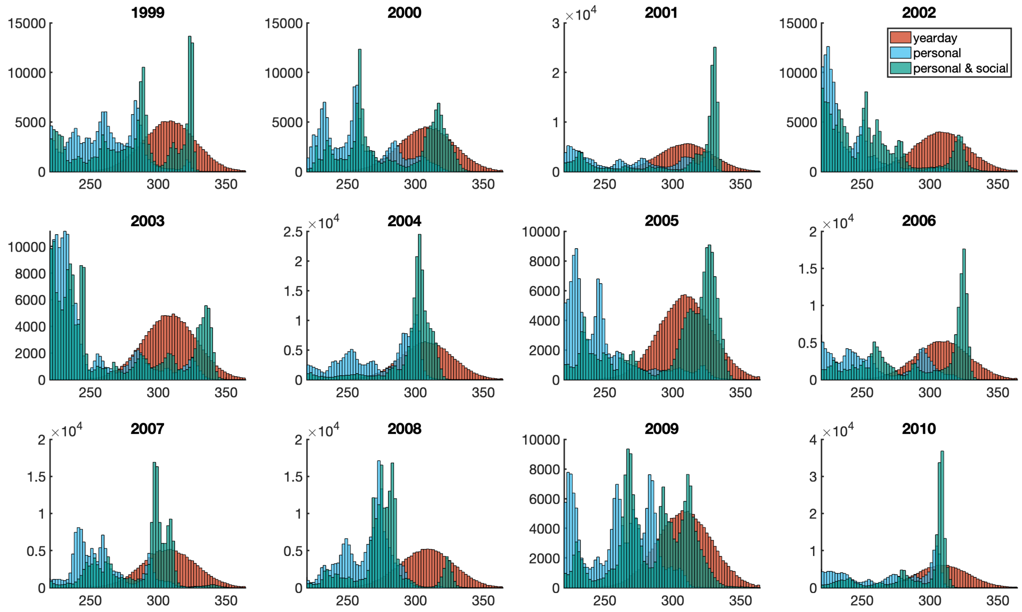

Supplementary Figure S 8: **Yearly migration distributions.** Representative migration distributions of personal, personal & social, and day of year migration strategies. Histograms show the yearday of migration initiation. Migration distributions shown for the full population. Data compiled from 100 simulations for each year, with 2,000 agents per simulation.

to transiting behaviors between September and October. On the other hand, the personal and social model shows a population level swtich from foraging to transiting behaviors between October and November, consistent with the empirical Monterey Bay hydrophone findings (Figure 2C; Oestreich et al., 2020).

8.2 Call Radius

Figure 11 provides additional insight on the impact of the maximum call radius. Migration distributions for three years of the personal & social strategy are show in Figure 11a. Across the three years, we see convergence of the median migration dates and a narrowing of the interquartile range, indicating an increased call radius leads to a more collective migration (additionally supported by Figure 11b).

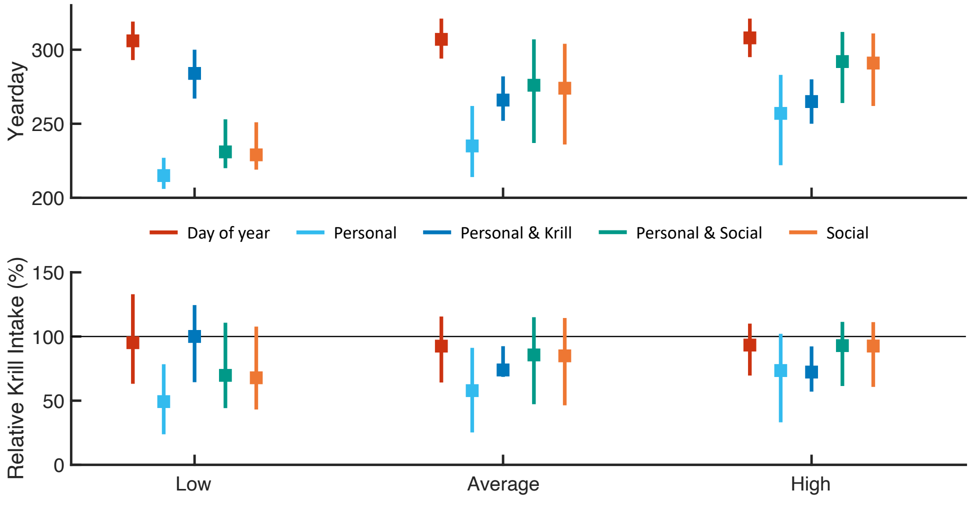

Supplementary Figure S 9: **Southward migrations by krill availability.** Southward migration distributions and krill intake for modeled populations separated by krill availability. Boxplots show (a) IQR of migration distributions and (b) relative krill intake for each migration mechanism. Values in (b) are computed as a percentage of the total non-migratory (null) population intake. Grey line indicates the median intake of the null population. Results from years 1990-2010 aggregated by yearly krill availability.

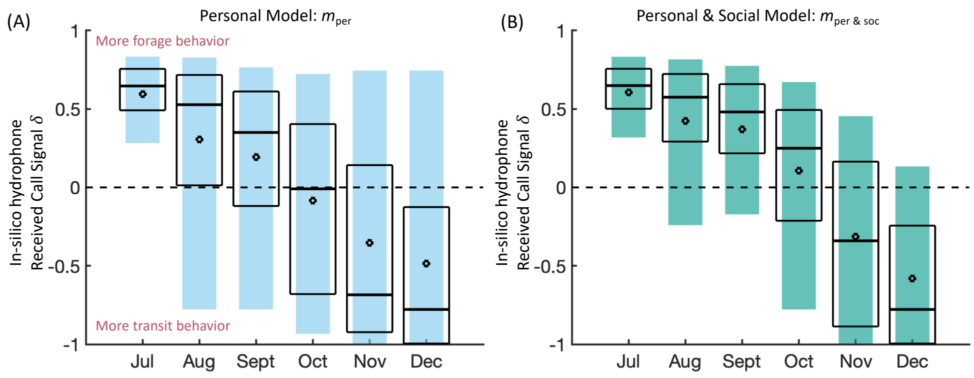

Supplementary Figure S 10: **In-silico hydrophone recordings.** Call signals received by the in-silico hydrophone across years 1990-2010, binned by month. Data for (A) the personal model and (B) the personal and social model. Boxes show the median values and 25-75% quartiles. Shaded bars indicate the 10-90% extent of the data, mean values are indicated by circles.

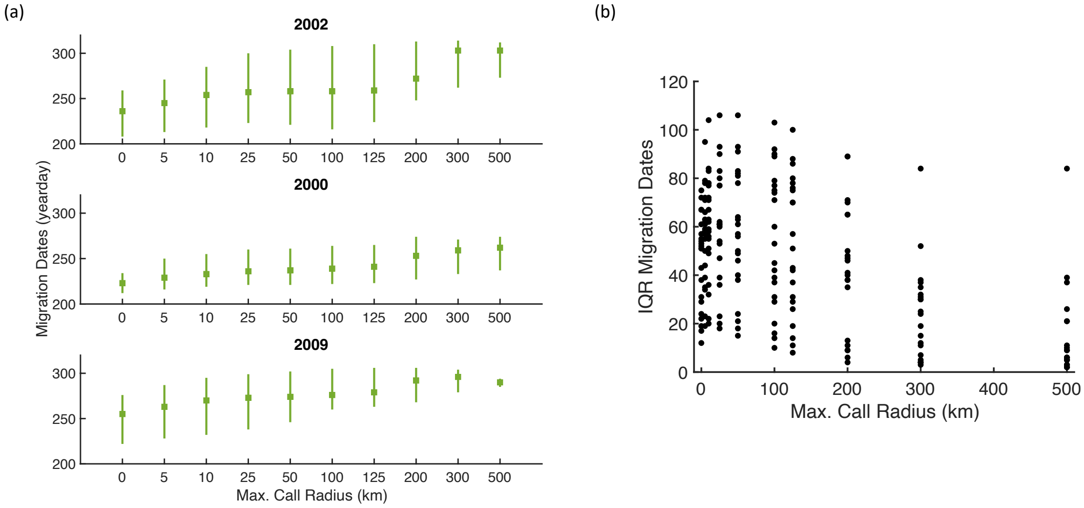

Supplementary Figure S 11: **Impact of maximum call radius.** (a) Representative yearly boxplots of southward migration distributions as a function of maximum call radius. (b) Interquartile range (IQR) of migration dates as a function of maximum call radius. Data from years 1990-2010. Both sets of results are from the personal & social model.

References

Abrahms, B., Hazen, E. L., Aikens, E. O., Savoca, M. S., Goldbogen, J. A., Bograd, S. J., Jacox, M. G., Irvine, L. M., Palacios, D. M., & Mate, B. R. (2019). Memory and resource tracking drive blue whale migrations. *Proceedings of the National Academy of Sciences*, *116*(12), 5582–5587. https://doi.org/10.1073/pnas.1819031116

Bailey, H., Mate, B., Palacios, D., Irvine, L., Bograd, S., & Costa, D. (2009). Behavioural estimation of blue whale movements in the northeast pacific from state-space model analysis of satellite tracks. *Endangered Species Research*, *10*, 93–106.

Dodson, S., Abrahms, B., Bograd, S. J., Fiechter, J., & Hazen, E. L. (2020). Disentangling the biotic and abiotic drivers of emergent migratory behavior using individual-based models. *Ecological Modelling*, *432*, 109225. https://doi.org/10.1016/j.ecolmodel.2020.109225

Fiechter, J., Santora, J. A., Chavez, F., Northcott, D., & Messié, M. (2020). Krill Hotspot Formation and Phenology in the California Current Ecosystem. *Geophysical Research Letters*, *47*(13), e2020GL088039. https://doi.org/10.1029/2020gl088039

Grimm, V., Berger, U., Bastiansen, F., Eliassen, S., Ginot, V., Giske, J., Goss-Custard, J., Grand, T., Heinz, S. K., Huse, G., Huth, A., Jepsen, J. U., Jørgensen, C., Mooij, W. M., Müller, B., Pe'er, G., Piou, C., Railsback, S. F., Robbins, A. M., … DeAngelis, D. L. (2006). A standard protocol for describing individual-based and agent-based models. *Ecological Modelling*, *198*(1-2), 115–126. https://doi.org/10.1016/j.ecolmodel.2006.04.023

Grimm, V., Berger, U., DeAngelis, D. L., Polhill, J. G., Giske, J., & Railsback, S. F. (2010). The ODD protocol: A review and first update. *Ecological Modelling*, *221*(23), 2760–2768. https://doi.org/10.1016/j.ecolmodel.2010.08.019

Grimm, V., Railsback, S. F., Vincenot, C. E., Berger, U., Gallagher, C., DeAngelis, D. L., Edmonds, B., Ge, J., Giske, J., Groeneveld, J., Johnston, A. S. A., Milles, A., Nabe-Nielsen, J., Polhill, J. G., Radchuk, V., Rohwäder, M.-S., Stillman, R. A., Thiele, J. C., & Ayllón, D. (2020). The ODD Protocol for Describing Agent-Based and Other Simulation Models: A Second Update to Improve Clarity, Replication, and Structural Realism. *Journal of Artificial Societies and Social Simulation*, *23*(2). https://doi.org/10.18564/jasss.4259

Irvine, L. M., Mate, B. R., Winsor, M. H., Palacios, D. M., Bograd, S. J., Costa, D. P., & Bailey, H. (2014). Spatial and Temporal Occurrence of Blue Whales off the U.S. West Coast, with Implications for Management (A. Fahlman, Ed.). *PLoS ONE*, *9*(7), e102959–10. https://doi.org/10.1371/journal.pone.0102959

Mann, H. B., & Whitney, D. R. (1947). On a test of whether one of two random variables is stochastically larger than the other. *The Annals of Mathematical Statistics*, *18*(1), 50–60. Retrieved June 7, 2023, from http://www.jstor.org/stable/2236101

Marino, S., Hogue, I. B., Ray, C. J., & Kirschner, D. E. (2008). A methodology for performing global uncertainty and sensitivity analysis in systems biology. *J. Theor. Biol.*, *254*(1), 178–196. https://doi.org/10.1016/j.jtbi.2008.04.011

Oestreich, W. K., Abrahms, B., McKenna, M. F., Goldbogen, J. A., Crowder, L. B., & Ryan, J. P. (2022). Acoustic signature reveals blue whales tune life-history transitions to oceanographic conditions. *Functional Ecology*, *36*(4), 882–895. https://doi.org/10.1111/1365-2435.14013

Oestreich, W. K., Fahlbusch, J. A., Cade, D. E., Calambokidis, J., Margolina, T., Joseph, J., Friedlaender, A. S., McKenna, M. F., Stimpert, A. K., Southall, B. L., Goldbogen, J. A., & Ryan, J. P. (2020). Animal-Borne Metrics Enable Acoustic Detection of Blue Whale Migration. *Current Biology*, *30*(23), 4773–4779.e3. https://doi.org/10.1016/j.cub.2020.08.105

The MathWorks Inc. (2022a). *Matlab version: 9.13.0 (r2022b)*. Natick, Massachusetts, United States. https://www.mathworks.com

The MathWorks Inc. (2022b). *Parallel computing toolbox version: 7.7 (r2022b)*. Natick, Massachusetts, United States. https://www.mathworks.com

Virtanen, P., Gommers, R., Oliphant, T. E., Haberland, M., Reddy, T., Cournapeau, D., Burovski, E., Peterson, P., Weckesser, W., Bright, J., van der Walt, S. J., Brett, M., Wilson, J., Millman, K. J., Mayorov, N., Nelson, A. R. J., Jones, E., Kern, R., Larson, E., … SciPy 1.0 Contributors. (2020). SciPy 1.0: Fundamental Algorithms for Scientific Computing in Python. *Nature Methods*, *17*, 261–272. https://doi.org/10.1038/s41592-019-0686-2
